# Supplementary material for: Quantitative Analysis of Fission Yeast Transcriptomes and Proteomes in Proliferating and Quiescent Cells
Source: Cell. 2012 Oct 26;151(3):671–83. doi: 10.1016/j.cell.2012.09.019 (PMC3482660; doi:10.1016/j.cell.2012.09.019)
Supplement: Document S2. Article plus Supplemental Information [file mmc2.pdf]

Cell

Quantitative Analysis of Fission Yeast Transcriptomes and Proteomes in Proliferating and Quiescent Cells

Samuel Marguerat,[1] Alexander Schmidt,[2] Sandra Codlin,[1] Wei Chen,[3] Ruedi Aebersold,[4,5,*] and Jürg Bähler[1,*]
[1]University College London, Department of Genetics, Evolution and Environment and UCL Cancer Institute, London WC1E 6BT, UK
[2]Proteomics Core Facility, Biozentrum, University of Basel, CH-4056 Basel, Switzerland
[3]The Berlin Institute for Medical Systems Biology, Max-Delbrück-Centrum für Molekulare Medizin (MDC), 13092 Berlin, Germany
[4]Department of Biology, Institute of Molecular Systems Biology, ETH Zurich, CH-8093 Zurich, Switzerland
[5]Faculty of Science, University of Zurich, CH-8057 Zurich, Switzerland
*Correspondence: aebersold@imsb.biol.ethz.ch (R.A.), j.bahler@ucl.ac.uk (J.B.)
http://dx.doi.org/10.1016/j.cell.2012.09.019

SUMMARY

Data on absolute molecule numbers will empower the modeling, understanding, and comparison of cellular functions and biological systems. We quantified transcriptomes and proteomes in fission yeast during cellular proliferation and quiescence. This rich resource provides the first comprehensive reference for all RNA and most protein concentrations in a eukaryote under two key physiological conditions. The integrated data set supports quantitative biology and affords unique insights into cell regulation. Although mRNAs are typically expressed in a narrow range above 1 copy/cell, most long, noncoding RNAs, except for a distinct subset, are tightly repressed below 1 copy/cell. Cell-cycle-regulated transcription tunes mRNA numbers to phase-specific requirements but can also bring about more switch-like expression. Proteins greatly exceed mRNAs in abundance and dynamic range, and concentrations are regulated to functional demands. Upon transition to quiescence, the proteome changes substantially, but, in stark contrast to mRNAs, proteins do not uniformly decrease but scale with cell volume.

INTRODUCTION

Gene regulation is crucial to implement genomic information and to shape properties of cells and organisms. Transcriptomes and proteomes are dynamically tuned to the requirements of cell volume, physiology and external factors. Although transcriptomic and proteomic approaches have provided ample data on relative expression changes between different conditions, little is known about actual numbers of RNAs and proteins within cells and how gene regulation affects these numbers. More generally, most data in biology are qualitative or relatively quantitative, but ultimately many biological processes will only be understood if investigated with absolute quantitative data to support mathematical modeling. Other areas of science have long appreciated the limits of relative, or compositional, data and potential pitfalls of their naive analysis (Lovell et al., 2011).

Insights into numbers and cell-to-cell variability of selected mRNAs and proteins have been provided by single-cell studies (Larson et al., 2009), but these approaches require genetic manipulation and are not well suited for genome-scale analyses. Relating mRNA to protein abundance in single cells is challenging, with only one such study available for a prokaryote (Taniguchi et al., 2010). Global mRNA abundance for yeast populations have been estimated (Holstege et al., 1998; Miura et al., 2008). There are no comparisons for cellular concentrations of mRNAs and the emerging diversity of noncoding RNAs.

RNA-seq now allows actual counting of RNA numbers, offering unbiased genome-wide information on average cellular RNA concentrations in cell populations (Ozsolak and Milos, 2011). Moreover, the global quantification of proteins has recently become possible owing to advances in mass spectrometry, giving valuable insight into the protein content of different cells (Beck et al., 2011; Cox and Mann, 2011; Maier et al., 2011; Nagaraj et al., 2011; Vogel and Marcotte, 2012).

Here, we combine quantitative RNA-seq and mass spectrometry to analyze at unprecedented detail and scale how changes in cell physiology and volume are reflected in the cellular concentrations of all coding and noncoding RNAs and most proteins. We analyze two fundamental physiological states in fission yeast: (1) proliferating cells that need to constantly replenish their RNAs and proteins, and (2) postmitotic cells that do not grow or divide owing to nitrogen limitation and reversibly arrest in a quiescent state (Yanagida, 2009). Although quiescent states are common, both for yeast and for cells in the human body, most research has focused on proliferating cells. The ability to alternate between proliferation and quiescence is central to tissue homeostasis and renewal, pathophysiology, and the response to life-threatening challenges (Coller, 2011). For example, quiescent lymphocytes

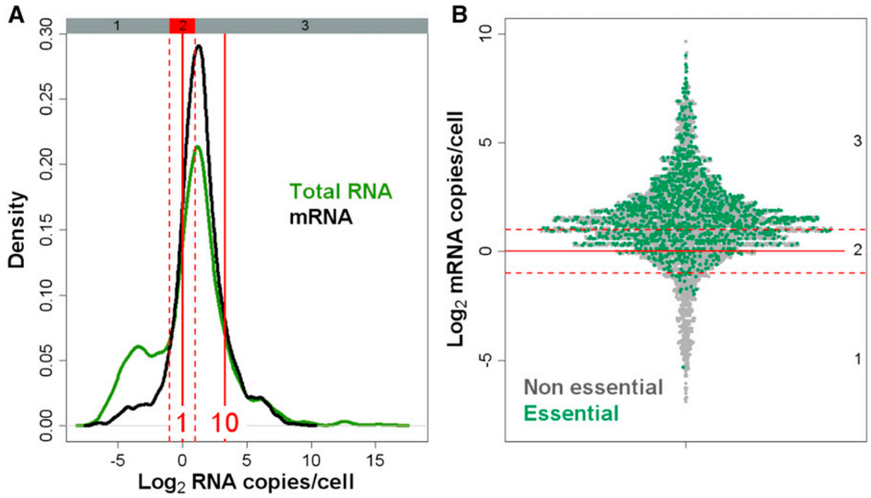

Figure 1. Transcriptome Quantification in Proliferating Cells

(A) Abundance distribution of total RNA (green) and mRNA (black). Red vertical lines indicate 1 and 10 RNA copies/cell, and red hatched lines delimit expression zones 1 to 3. See also Figure S1 and Table S10.

(B) Abundance for all detected mRNAs (each dot represents a gene). Green and gray dots correspond to essential and non essential genes, respectively. Expression zones are indicated at right.

and dermal fibroblasts become activated to mount immune responses or support wound healing, respectively. Adult stem cells also alternate between proliferating and quiescent states, and the deregulation of either state can cause complex pathologies such as cancer (Li and Clevers, 2010).

Our integrated transcriptomic and proteomic data, acquired in parallel under highly controlled conditions in a simple model, afford varied biological insights and reveal key principles of RNA and protein expression in proliferating and quiescent cells with broad relevance for other eukaryotes. This rich resource also provides a quantitative framework toward a systems-level understanding of genome regulation, and the common units of the absolute data allow direct comparison of different biological processes and organisms.

RESULTS AND DISCUSSION

Transcriptome and Proteome Quantification in Two Conditions

We acquired quantitative expression data relative to absolutely calibrated standards for transcriptomes and proteomes of haploid fission yeast cells. For transcripts, genome-wide measurements were obtained by calibrating RNA-seq data from total RNA preparations with data on absolute cellular concentrations for 49 mRNAs, covering the dynamic expression range. The overall measurement error was estimated to be ~2-fold or less (Figure S1; Tables S1–S4 available online). Protein quantification was performed on the same cell samples using a mass spectrometry (MS) approach (Schmidt et al., 2011). Selected proteotypic peptides from 39 proteins (Table S5), covering the dynamic expression range, were used to absolutely quantify the corresponding proteins (Tables S6 and S7). These data were then used to translate the MS-intensities for the other proteins into estimates of cellular concentration (Figures S2A–S2D and S3; and Tables S8 and S9). The mean overall measurement error was estimated at 2.4- and 2.7-fold for proliferating and quiescent cells, respectively.

We quantified transcriptomes and proteomes in two distinct physiological conditions: (1) exponentially proliferating cells in defined minimal medium, and (2) quiescent cells, 24 hr after nitrogen removal (Figure S4). We first report the results from proliferating cells, and then relate our findings to corresponding data from quiescent cells. Table S4 provides the cellular copy numbers for RNAs and proteins in the two conditions.

Most mRNAs Are Expressed in Narrow Range above 1 Copy/Cell

In proliferating cells, we measured a total of ~41,000 mRNA molecules/cell on average, representing ~5% of the overall ~802,000 rRNAs/cell in our samples. Protein-coding genes produced a median of 2.4 mRNA copies/cell, ranging from ~0.01 to >810 copies (Figure 1A). Only 71 genes showed no detectable mRNA signal, 43 of which are annotated as "dubious" or "orphan" (Wood et al., 2012). To discuss our findings, we distinguished three somewhat arbitrary expression zones, set relative to the one RNA copy/cell mark (Figure 1A). Zone 1 contained low-abundance mRNAs detected at <0.5 copies/cell. Zone 2 mRNAs were expressed at ~1 copy/cell (0.5–2 copies), where fluctuations due to cell division or stochastic expression will strongly affect the presence of mRNAs in cells. Zone 3 mRNAs showed more robust expression at >2 copies/cell. Most mRNAs were expressed within a low and narrow range: whereas >90% of all annotated mRNAs (4,608/5,110) belonged to zones 2 or 3, 86.1% of these mRNAs were present at <10 copies/cell (Figure 1A). Low overall mRNA concentrations have also been reported for budding yeast, which has comparable gene numbers and cell size, with even lower estimates for median mRNA abundance (<1 copy/cell) and total mRNA molecules/cell (Holstege et al., 1998; Miura et al., 2008). Our findings are in line with a single-cell study of budding yeast, where five mRNAs show 2.6–13.4 copies/cell, with a total estimate of 60,000 mRNA molecules/cell (Zenklusen et al., 2008).

We examined the mRNAs of the 1,273 genes essential for growth (Kim et al., 2010), which are expected to be expressed in proliferating cells. Nearly all essential mRNAs were expressed in zones 2 or 3 (98.4%; Figure 1B). This finding raises the possibility that ~1 mRNA copy/cell defines a natural minimal threshold for productive gene expression.

The view of ~1 mRNA copy/cell as an expression threshold is supported by recent data from metazoa, where mRNA levels

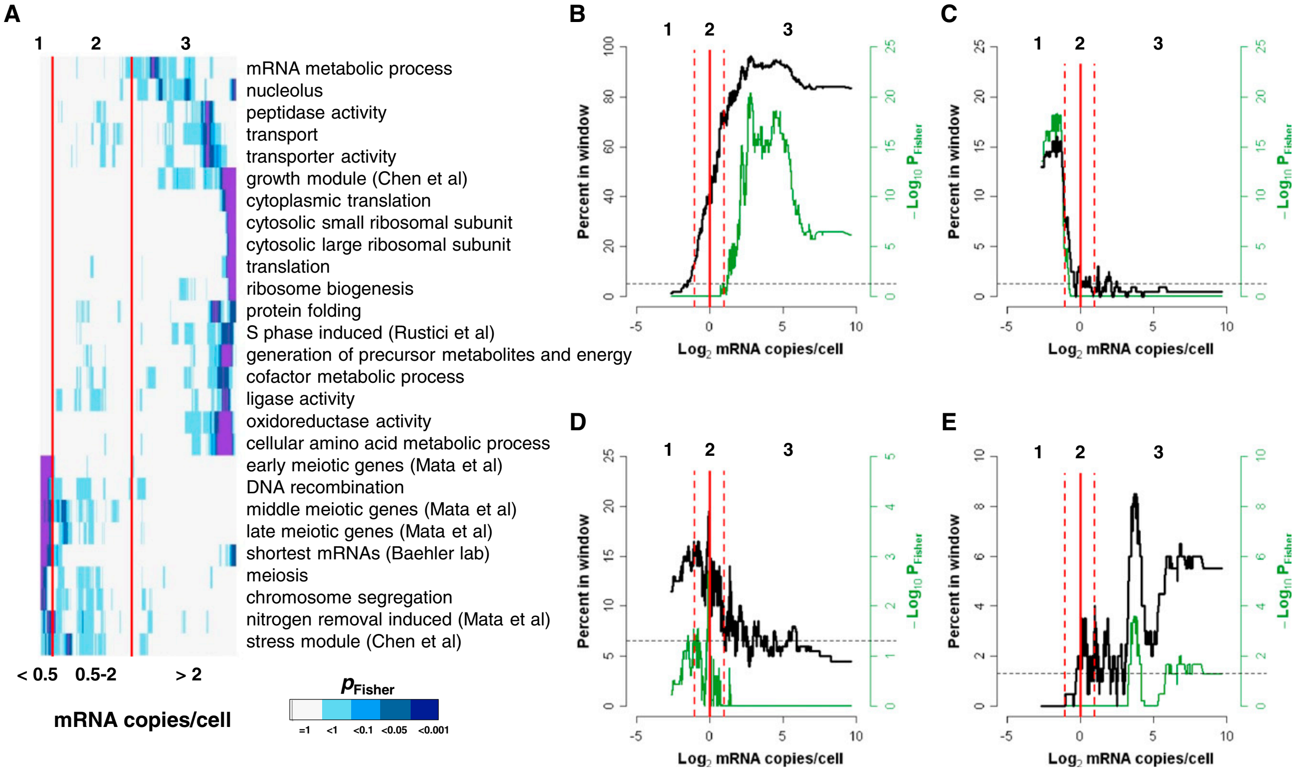

Figure 2. Functional Categories and Expression Zones

(A) Hierarchical clustering of p values (Fisher exact test, color-coded as indicated) assessing significance of overlap between genes in functional categories (rows) and 200-gene sliding windows of mRNA abundance (columns). Vertical red lines delimit the expression zones. Functional categories with p values <0.01 in ≥1 window are shown. See also Table S11.

(B) Frequency of genes for which corresponding protein is detected in 200-gene sliding window of mRNA abundance (black curve; left axis), together with p values (Fisher exact test) for significance of overlap between gene list and window (green curve; right axis).

(C) As in (B) for early meiotic differentiation genes (Mata et al., 2002).

(D) As in (B) for core environmental stress response genes (Chen et al., 2003).

(E) As in (B) for "protein folding" genes (Gene Ontology ID: 0006457).

show a bimodal distribution (Hebenstreit et al., 2011): one group of putative nonfunctional mRNAs present at <1 copy/cell, and another group of actively transcribed mRNAs expressed at >1 copy/cell. mRNA levels did not show such a bimodal distribution in fission yeast (Figure 1A). This disparity highlights that in differentiated metazoan cells many genes are not expressed, whereas in proliferating yeast cells most genes are actively expressed. Notably, when including long noncoding RNAs, which were mostly present at low abundance, fission yeast also showed a bimodal distribution for transcript levels (Figure 1A).

Characteristics of Three mRNA Expression Zones

Each expression zone was enriched for distinct functional categories (Figure 2A), revealing that genes participating in similar processes typically coordinate their cellular mRNA concentrations. The mRNA expression zones reflect protein expression as the 3,397 proteins detected in proliferating cells showed a strong bias toward highly expressed mRNAs (Figure 2B), although proteins of low abundance were also confidently detected (see below).

Only 431 genes were present in zone 1 (8.4% of 5,110 protein-coding genes), which were enriched for meiotic differentiation functions such as recombination and sporulation (Figures 2A and 2C). Genes induced during meiosis are tightly repressed during proliferation, and their expression is regulated at multiple levels including chromatin (Zofall et al., 2012), transcription (Mata et al., 2007), and mRNA turnover (Harigaya and Yamamoto, 2007; McPheeters et al., 2009). Only 31 (7.2%) of these genes produced detectable proteins, most of which were stress response genes and present near the upper limit of zone 1 (0.5 copies/cell). These findings support the notion that zone 1 genes are not actively transcribed and typically do not lead to productive protein expression. We propose that the presence of mRNAs at well below 1 copy/cell reflects active repression of the corresponding genes. Such low mRNA copy numbers could be the result of rare stochastic transcription (Hebenstreit et al., 2011).

The 1,664 genes of zone 2 included 27.8% of all essential genes, and 880 (52.9%) of these genes produced detectable proteins. These findings indicate that low mRNA concentrations (~1 copy/cell) are compatible with productive gene

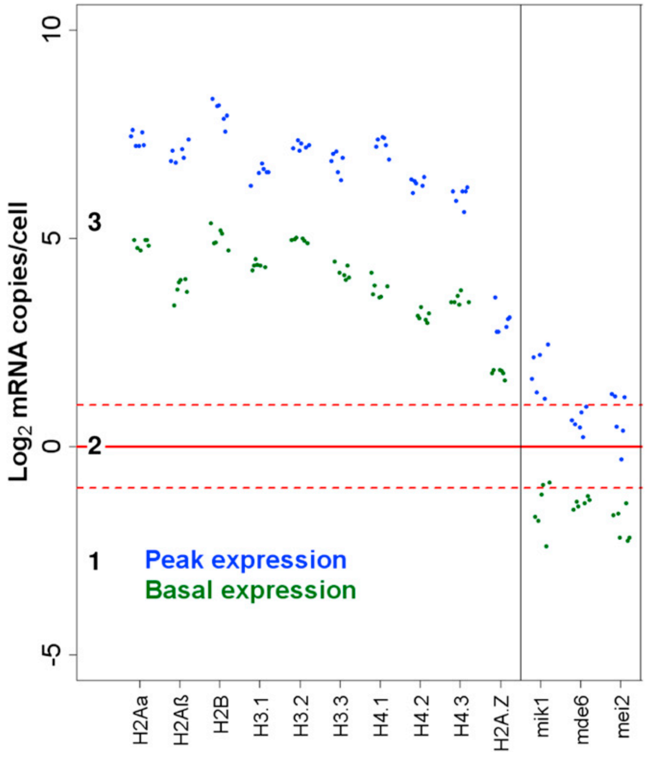

Figure 3. mRNA Copy Number Changes during Cell Cycle

Peak (blue) and basal (green) mRNA abundance of cell-cycle-regulated genes extrapolated from average data in asynchronous cultures, with 10% of cell-cycle assumed as duration for peak expression. Data for six cell-cycle time course experiments are indicated by clustered dots (Rustici et al., 2004). Left: ten histone mRNAs peaking during S phase; right: *mik1*, *mde6*, and *mei2* mRNAs peaking during M and G1 phases.

See also Figure S5 and Table S12.

expression. Zone 2 genes were functionally enriched for chromosome segregation, nitrogen starvation, and core environmental stress response (Figures 2A and 2D). The latter genes are rapidly induced in multiple stresses (Chen et al., 2003) and show highly variable expression across different experimental conditions (Pancaldi et al., 2010). This enrichment suggests that ~1 mRNA copy/cell corresponds to the basal expression typical of many stress response genes (Chen et al., 2003). Unlike the tight repression of meiotic genes, the basal expression of stress genes could enable a rapid response to sudden environmental challenges. Zone 2 was transitional between zones 1 and 3 also with respect to protein detection (Figure 2B). We propose that low basal mRNA expression might not always lead to robust protein expression but might maintain a responsive chromatin environment, e.g., for genes that require rapid upregulation during stress. Moreover, such low average expression could reflect a "bet-hedging" strategy to diversify cellular phenotypes and promote population survival to unexpected environmental challenges (López-Maury et al., 2008).

Zone 3 contained 2,944 genes (57.6% of all genes), which were enriched for several functional categories (Figure 2A). For example, genes involved in translation and protein folding tended to be highly expressed (Figure 2E). Proteins were detected for 2,486 (84.4%) of the zone 3 genes, indicating that robust mRNA expression typically results in robust protein expression.

Together, these data show that mRNAs of different functional categories are typically expressed in distinct abundance ranges. The data further support the notion that an expression of ~1 mRNA copy/cell defines a minimal threshold for productive gene expression. We conclude that the three mRNA expression zones reflect characteristic gene groups with respect to regulation, cellular functions, and protein production.

Effect of Cell-Cycle-Regulated Gene Expression on mRNA Numbers

Global studies have revealed hundreds of fission yeast genes that are periodically expressed during the cell cycle (Marguerat et al., 2006). The corresponding mRNA copy numbers will therefore fluctuate, and our quantitative data from asynchronous cell cultures reflect time-averaged mRNA counts. The effects of cell-cycle-regulated gene expression on absolute mRNA abundance are not known. Two scenarios are plausible: periodic gene expression might boost mRNA numbers for proteins required at higher levels during certain cell-cycle phases, or it might act in a switch-like manner to tightly restrict expression to a specific phase.

To distinguish between these two hypotheses, we applied simple modeling to extrapolate absolute changes in mRNA abundance of cell-cycle-regulated genes from our data in asynchronous cultures. The model assumes that periodic genes peak in expression during a defined cell-cycle phase and show basal expression during the other phases. We derived phase-specific mRNA copy numbers for 241 periodic genes with expression peaking in M, G1, or S phase (Figure 3). Most of these genes (96.3%) showed variations in mRNA expression that remained within zones 2 and 3 throughout the cell cycle. For example, the mRNAs for 10 histone genes were abundant throughout the cell cycle, with their numbers peaking during DNA replication (Figure 3). This pattern is consistent with the idea that periodic gene expression boosts mRNA numbers to accommodate an increased demand for histones during S phase, with a high basal requirement in other phases.

Only nine genes showed a more switch-like pattern of transcription: they belonged to zones 2 or 3 during peak expression, but dropped to zone 1 during basal expression, thus crossing the ~1 mRNA copy/cell threshold (Figure 3). We propose that expression of these genes is restricted to a specific cell-cycle phase, and repressed when they may be harmful. For example, the *mik1* gene encodes an inhibitor of mitosis with a tightly restricted expression window at both mRNA and protein levels (Ng et al., 2001). Another example was *mei2*, encoding a protein that promotes untimely meiosis when activated at the wrong time (Harigaya and Yamamoto, 2007). We conclude that periodic gene expression generally tunes mRNA numbers to specific requirements in different cell-cycle phases but also, in special cases, reflects regulatory switches restricting the expression of critical regulators to specific phases.

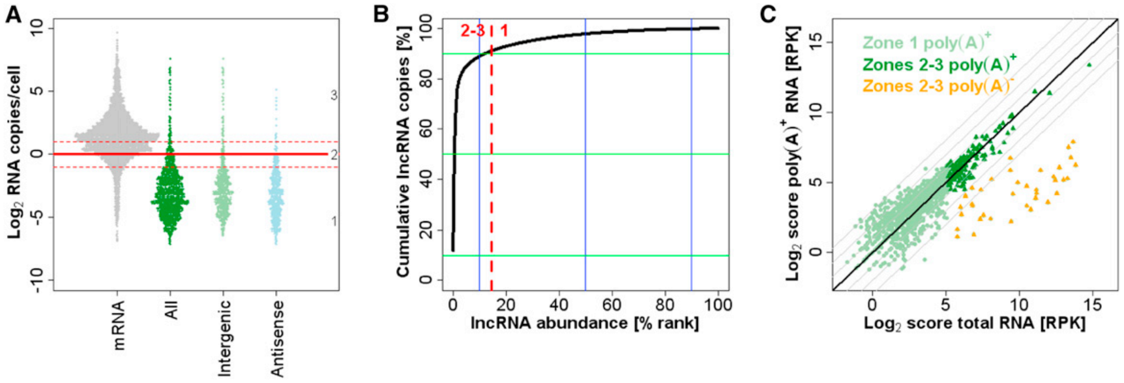

Figure 4. Quantitative Analysis of Long Noncoding RNAs

(A) Absolute abundance of mRNAs (gray), and all (dark green), intergenic (bright green), and antisense (blue) lncRNAs. Expression zones are indicated at right.
(B) Cumulative plot of copy numbers contributed by lncRNA genes ranked by decreasing abundance, with genes expressed in zones 2 and 3 at left of red line.
(C) Sequence scores for lncRNAs in libraries made from total versus poly(A)$^+$ RNA. Bright green circles, lncRNAs expressed in zone 1; dark green and orange triangles, lncRNAs expressed in zones 2 or 3; orange, lncRNAs that are $\geq$4-fold more abundant in total than in poly(A)$^+$ RNA library.
See also Table S13.

Long Noncoding RNAs Are Typically Present below 1 Copy/Cell

Substantial transcriptional activity occurs outside of protein-coding genes and produces distinct noncoding RNAs. Besides the well known RNAs involved in gene expression such as rRNAs, tRNAs, snRNAs, and snoRNAs (Figure S1), 1,557 long noncoding RNAs (lncRNAs) have been identified in fission yeast (Rhind et al., 2011; Wilhelm et al., 2008). These lncRNAs are reminiscent of lincRNAs in multicellular eukaryotes and unannotated transcripts in budding yeast (SUTs, CUTs, XUTs) (Atkinson et al., 2012), but differ from the short RNAs produced by RNA interference pathways (Grewal, 2010). In proliferating cells, we could quantify 86.4% (1,346/1,557) of these lncRNAs, which together accounted for only 1,672 RNA molecules/cell (Table S10). Accordingly, 1,159 (85.5%) of these lncRNAs belonged to zone 1, numbering well below 1 copy/cell, similar to tightly repressed mRNAs (Figures 1A and 4A). lncRNAs transcribed both in intergenic regions and antisense to coding genes typically belonged to zone 1 (Figure 4A). By analogy with meiotic genes, such low abundance could reflect tight repression at transcriptional, posttranscriptional, and/or chromatin levels. The remaining 187 lncRNAs (14.5%) were expressed in zones 2 and 3, at ~1–200 copies/cell. Notably, this small group accounted for >90% of the total cellular number of lncRNA molecules (Figure 4B). This group was not enriched for lncRNAs conserved in other fission yeast species (Rhind et al., 2011). The coding genes that were associated with antisense RNAs expressed in zones 2 or 3 were more likely to be repressed in zone 1 ($p_{binomial} < 10^{-8}$), consistent with a role of antisense transcription in repressing the corresponding sense transcription.

We compared the sequence scores from RNA-seq libraries produced from either total or poly(A)-enriched RNA. Most lncRNAs were present at similar levels in the two libraries, irrespective of their abundance (Figure 4C). This result suggests that most lncRNAs are polyadenylated and therefore likely transcribed by RNA polymerase II (Pol II). Intriguingly, 38 lncRNAs were much more abundant in the total RNA library (Figure 4C). These lncRNAs were depleted during poly(A) enrichment and hence likely not polyadenylated. This finding raises the possibility that these lncRNAs are not transcribed by Pol II, or that they are matured via poly(A) trimming (Lemay et al., 2010). These lncRNAs showed no particular sequence features using Rfam (Gardner et al., 2011), and they were not similar to any well-known RNAs such as snRNAs or snoRNAs. Remarkably, although these lncRNAs made up only 2.4% of the known lncRNA repertoire, they accounted for 63.6% of the total lncRNA molecules. Taken together, this analysis uncovered two distinct classes of lncRNAs that differ based on their absolute expression and polyadenylation status, with a small class of nonpolyadenylated lncRNAs contributing the majority of all cellular lncRNA molecules.

Proteins Greatly Exceed mRNAs in Abundance and Dynamic Range

In proliferating cells, we could quantify 3,397 (66.5%) of the 5,110 predicted proteins, adding up to an average of 60.3 million protein molecules/cell. The identified proteins showed no strong bias against any protein class (Figure S2E), underlining the broad coverage achieved. Protein-coding genes produced a median of 3,919 protein copies/cell, with a dynamic range of five orders of magnitude (Figure 5A). The most abundant protein was the translation factor EF-1α (Tef102), expressed at ~1.1 million copies/cell, whereas the lowest detectable protein was the formin Cdc12, expressed at <100 copies/cell. Our data were similar to quantitative microscopy data for 27 cytokinesis proteins (Figure S2H) (Wu and Pollard, 2005). On average, proteins were ~1,850 times more abundant than their respective mRNAs. This finding indicates that translation serves as a global amplification step, although some of this difference could also reflect longer half-lives for proteins than for mRNAs.

The mRNAs coding for the 3,397 detected proteins were greatly enriched in expression zones 2 and 3 (Figure 5B). Moreover, the 1,273 essential genes (Kim et al., 2010) produced a significantly higher proportion of detectable proteins (81.9%,

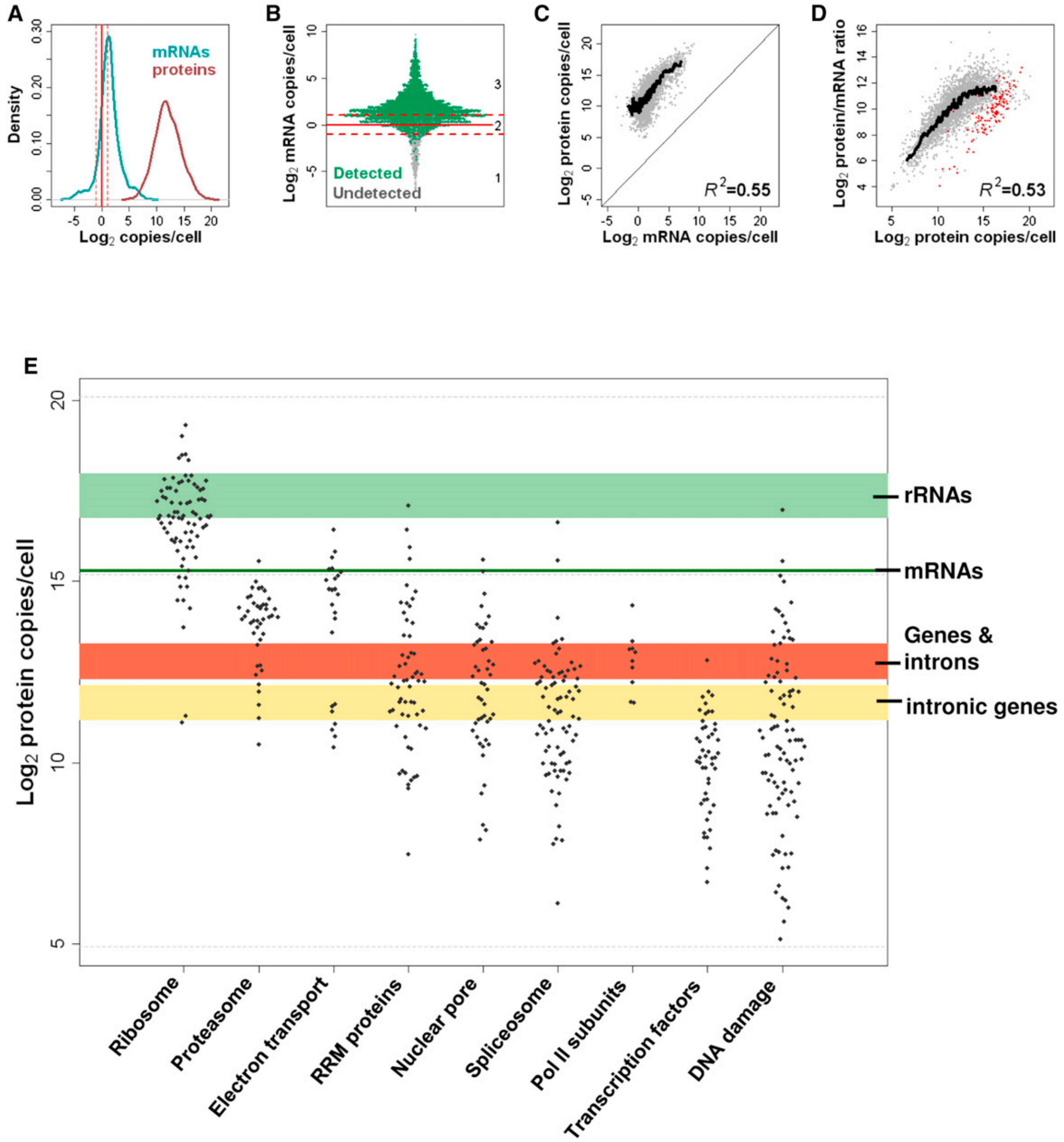

Figure 5. Quantitative Analysis of Proteome in Proliferating Cells

(A) Abundance distribution for mRNAs (green) and proteins (red). Red vertical lines delimit expression zone 2 (0.5–2 mRNA copies/cell). See also Figures S2, S3, and Table S10.

(B) Absolute abundance for all mRNAs (each dot represents a gene). Dark and light blue dots correspond to genes for which proteins were detected or not, respectively.

(C) Protein versus mRNA abundance. Black curve, sliding median.

(D) Protein/mRNA ratio versus protein abundance. Red dots: ribosomal proteins; black curve: sliding median.

(E) Protein abundance for selected functional categories. Each dot represents a protein. Haploid *Schizosaccharomyces pombe* cells contain 5,110 and 10,220 annotated protein-coding genes in G1 and G2 phase, respectively (red zone), and 5,348 introns across 2,523 intron-containing genes (red and yellow zones, respectively). In proliferating cells, we measured ~41,000 mRNA molecules (dark green line) and 1.1–2.6 × 10^5 copies of each rRNA (green zone). Ribosomal proteins copies/cell for paralogs were summed up.

See also Figure S6.

$p_{binomial} < 10^{-15}$). The 458 robustly expressed zone 3 mRNAs not associated with detectable proteins were enriched for mRNAs upregulated during mitosis and for cell surface functions (although protein detection was not affected by numbers of *trans*-membrane domains; Figure S2F; Table S11). Proteins encoded by mitotic mRNAs may only be expressed during a short

cell-cycle window, and thus fall below the detection limit in unsynchronized cells. Accordingly, of eight cell-cycle-regulated proteins tested, only two were detectable by fluorescence microscopy, and they showed expression restricted to specific phases (Table S12). Small proteins typically had less than or equal to five MS-compatible peptides and showed lower identification rates (Figure S2G; Table S11). Taken together, these data indicate that the proportion of proteins not detected due to technical limitation (rather than lack of expression) was <20% of the expressed proteome. Thus, we provide accurate absolute quantification for most fission yeast proteins, and these proteins substantially exceed the mRNAs in abundance and dynamic range.

Coordinated Expression at mRNA and Protein Levels

Copy numbers of mRNAs and corresponding proteins were highly correlated (Figure 5C). This global relationship between transcriptome and proteome means that mRNA levels largely reflect the respective protein levels. Translational properties of mRNAs, such as ribosome numbers and densities (Lackner et al., 2007), were also correlated with protein abundance (R^2 ~0.1). These data extend previous observations that gene expression is coordinated at the levels of transcription, mRNA decay and translation (Lackner et al., 2007) to now also include protein abundance. However, the ratios between protein and corresponding mRNA copy numbers spanned over three orders of magnitude, ranging from 14 to 61,060. This result points to substantial regulation at the levels of translation and/or protein turnover. The protein/mRNA ratios were also strongly correlated with the corresponding protein numbers, but they saturated at higher protein levels (Figure 5D). This phenomenon suggests that translation becomes limiting for the most abundant proteins (e.g., owing to saturation of ribosomes on mRNAs), and that these proteins thus rely on relatively higher mRNA numbers to boost their abundance. Schwanhäusser et al. (2011) have observed a similar saturation when comparing 5,000 mouse protein levels to respective translation rates. Notably, the highly expressed ribosomal proteins formed a distinct group that showed significantly lower protein/mRNA ratios compared to genes with similar protein expression ($p_{Wilcoxon} < 10^{-9}$; Figure 5D). This observation, and related data from Schmidt et al. (2007), suggests that ribosomal proteins rely more on mRNA levels than on translation for their high abundance. As ribosomal proteins act in a complex with rRNAs, the emphasis on transcriptional control might ensure better regulatory coordination with the nontranslated rRNAs. Other ribonucleoprotein complexes such as the spliceosome, however, did not show such lower protein/RNA ratios.

Protein abundance was negatively correlated with protein length (R^2 −0.07), consistent with shorter mRNAs being more efficiently translated (Lackner et al., 2007). This finding supports the idea that highly expressed proteins evolved more streamlined structures due to energetic constraints. Conversely, no correlation between mRNA abundance and protein length was evident (R^2 ~4×10^{-5}), suggesting that any regulatory adaptation occurred at the levels of translation and/or protein stability, which are energetically more costly than transcription.

Strikingly, the 20% most abundant proteins accounted for 81.3% of the total protein molecules in proliferating cells, and this skew was also reflected in the corresponding mRNA numbers, albeit less pronounced (Figure S2I). This finding evokes the Pareto principle (“20–80 rule”) of unequal distribution in economics and elsewhere, and it highlights that the cell invests most energy to produce many copies of relatively few proteins. In addition, the distribution of individual protein frequencies as a function of their expression rank fitted power-law distributions, extending a characteristic of mRNA expression to proteins (Figures S2J and S2K). Taken together, we conclude that gene regulation is globally coordinated and streamlined across the expression spectrum.

Protein Abundance in Context of Cellular Landmarks and Functions

We compared protein concentrations with cellular “landmarks” for meaningful biological context (Figure 5E). The ribosome is a large complex composed of single copies of multiple proteins and rRNAs. Thus, transcriptome and proteome data correctly calibrated relative to each other should arrive at similar estimates for total ribosome numbers, allowing cross-validation of our two independent data sets. Reassuringly, the numbers for most ribosomal proteins were consistent with the numbers for different rRNAs (Figure 5E), indicating that there are 1–2 × 10^5 ribosomes in an average proliferating cell. This number is comparable to an electron microscopy estimate (~5 × 10^5 ribosomes/cell; Maclean, 1965). For further confirmation, we calculated the total number of ribosomes associated with mRNAs by multiplying copy numbers of all individual mRNAs with their associated ribosome numbers obtained from polysome profiling (Lackner et al., 2007), resulting in a total of ~1.5 × 10^5 ribosomes/cell. Thus, several independent data point to similar cellular ribosome numbers, corroborating that our quantification of transcripts and proteins is accurate, both with respect to absolute numbers and relative to each other. Some ribosomal proteins showed much lower abundance, however, supporting the view that they may have nonribosomal functions (Bhavsar et al., 2010). The median mRNA and protein expression of single-copy ribosomal proteins was significantly higher than the median expression of duplicated ribosomal proteins (Figure S6A); this finding raises the possibility that paralogs contribute to only part of the ribosome pool, suggesting heterogeneous ribosome composition. Proliferating cells contained approximately four times more ribosomes than mRNAs, illustrating the amplification at the level of translation.

The proteasome is a large complex that degrades ubiquitinated proteins. An average cell contained 1–2 × 10^4 proteasomes, approximately ten times fewer than ribosomes (Figure 5E). This result highlights that more resources are invested in protein production than in protein degradation in proliferating cells that need to continuously produce new proteins to compensate for dilution from cell growth and division.

Proteins of the Pol II transcription complex were present at 7,780 median copies/cell, meaning that cells contain ~1 Pol II/gene (Figure 5E). This low estimate suggests that Pol II could become limiting, consistent with the finding that Pol II subunit mutants are haplo-insufficient (Kim et al., 2010), and with

evidence for transcription factories where limiting factors are concentrated for gene expression (Cook, 2010). One Pol II subunit, Rpc10, was more highly expressed (20,748 copies/cell), reflecting that Rpc10 is part of all three RNA polymerase complexes.

We also analyzed regulatory transcription factors (TFs) that direct Pol II to specific subsets of genes. The numbers of the detectable TFs ranged from ~100 to >7,000 copies/cell. TFs controlling meiotic differentiation (Mei4, Atf21, Atf31, Rep1; Mata et al., 2007) were not detected as proteins and showed low mRNA abundance in zone 1, whereas the heat shock factor Hsf1 was the most highly expressed TF (7,244 copies). The large dynamic range in TF abundance could reflect different mechanisms of transcriptional control, or TF copy numbers might scale with the numbers of their target genes, although they did not correlate with the occurrence of known DNA motifs (Figure S6B).

Proteins of the spliceosome complex were present at 2,675 median copies/cell, similar to the number of intron-containing genes (Figure 5E). Two splicing proteins, Snu13 and Uap56, were found at much higher numbers (~1 × 10^5 and 5 × 10^4 copies, respectively), probably reflecting their additional roles in rRNA maturation and mRNA export (Dobbyn and O'Keefe, 2004; Strässer and Hurt, 2001). Thus, the cell produces just enough spliceosomes to deal with the 2,523 intron-containing transcriptional units, supporting the view that most splicing occurs cotranscriptionally in a chromosomal context (de Almeida and Carmo-Fonseca, 2012).

Proteins with RNA-recognition motifs (RRM) are an important class of RNA-binding proteins that control posttranscriptional gene expression. Intriguingly, RRM protein abundance was 4-fold higher than TF abundance (Figure 5E), evocative of the 4-fold difference between mRNA molecules and protein-coding genes during G2-phase. Thus, the numbers of RRM proteins and TFs scale with the numbers of their respective binding partners. The detected RRM proteins showed large differences in abundance, ranging from only 175 copies for the methyltransferase Set1 to 139,690 copies, more than mRNA molecules, for the uncharacterized Vip1. As for TFs, RRM proteins with meiotic functions (Mug24, Spo5, Crp79, Mug28, Mde7) where tightly repressed during proliferation and not detected as proteins. These findings suggest that some RRM proteins have transient or specialized roles by targeting few specific transcripts, whereas others have more ubiquitous roles, in line with genome-wide binding data (Hogan et al., 2008). Accordingly, the cytoplasmic Pabp, which binds to poly(A) tails of all mRNAs, showed the second highest expression at 87,000 copies/cell. This result suggests that approximately two Pabp proteins bind to average mRNAs, in line with findings that poly(A) tails in yeast contain ~50 residues on average (Lackner et al., 2007) and every Pabp covers 27 adenine residues (Baer and Kornberg, 1983).

Protein Expression Reflects Cellular Function

We also analyzed protein copy numbers with respect to different functional categories. Discrete patterns of protein expression distributions were evident, with proteins of different functions being significantly enriched for distinct abundance ranges (Figure S7). Thus, proteins of similar functions are often expressed at similar copy numbers. Three general protein expression groups were apparent. Lowly expressed proteins (<5,000 copies/cell) were enriched for regulators of biological processes such as TFs and protein kinases, and for proteins involved in chromosome structure and DNA repair. An intermediate group of proteins, expressed at ~0.5–1 × 10^4 copies/cell, often functioned in RNA metabolism, including splicing, processing or degradation. Highly expressed proteins (~1 × 10^4–1.1 × 10^6 copies/cell) were enriched for functions related to translation, growth, and metabolism. We conclude that, like mRNAs, proteins functioning together or in related biological processes typically share similar expression levels, and these levels reflect cellular requirements for different tasks or complexes.

Transcriptome Shrinks Globally during Quiescence

To analyze quantitative RNA and protein changes in a distinct physiological state, we also acquired data from cells after 24 hr of nitrogen starvation. Upon nitrogen removal, cells stop growth, divide twice, and arrest as postmitotic, quiescent cells. These cells remain metabolically active by recycling nitrogen, become highly resistant to multiple stresses, and survive for months (Yanagida, 2009).

Quiescent cells are stubby compared to proliferating cells, showing a median volume reduction of ~40%–50% within 12 hr of nitrogen removal (Figures S4A–S4D). Strikingly, during the same period, the RNA mass is reduced by ~85% to that of proliferating cells (Figure S4E). We measured a total of 89,470 rRNAs/quiescent cell, representing merely 11.2% of the number in proliferating cells (Figure 6A). The protein-coding transcriptome showed a somewhat lower reduction, shrinking to 7,419 total mRNAs (18% of proliferating cells; Figures 6A and 6B). Taking into account their smaller volumes, quiescent cells contained ~19.6% rRNA and 31.3% mRNA compared to proliferating cells. The reduction in mRNA copy numbers was global, and remarkably coordinated, with abundance in proliferating and quiescent cells remaining highly correlated (Figure 6C). We conclude that quiescent cells rely on a substantially smaller transcriptome, both with respect to RNA abundance and concentration.

Nevertheless, most mRNAs were still expressed within zones 2 or 3 during quiescence (49.8% and 15%, respectively), but with a median of only 0.69 copies. Thus, although shrinking by 82% in number, mRNAs retained ~72% of the diversity in proliferating cells. Only 81 mRNAs (1.6% of all) were >2-fold more abundant in quiescent than in proliferating cells, whereas 4,266 mRNAs (83.5%) were ≥2-fold less abundant. Thus, quiescent cells harbor a diminished but diverse transcriptome, with the majority of mRNAs being expressed at only ~1 copy/cell. It is possible that low mRNA concentrations represent a more robust expression during quiescence when cells do not grow and divide, and mRNAs might be stabilized for long-term endurance (Pluskal et al., 2011).

Figure 6D compares median mRNA copy numbers for selected functional categories in proliferating and quiescent cells. Most categories were substantially downregulated in quiescence, whereas a few retained similar numbers, including stress response and sexual differentiation (Figure 6D). Furthermore, three highly expressed categories, all related to protein translation, were downregulated more than average, yet these mRNAs remained the most abundant with respect to absolute

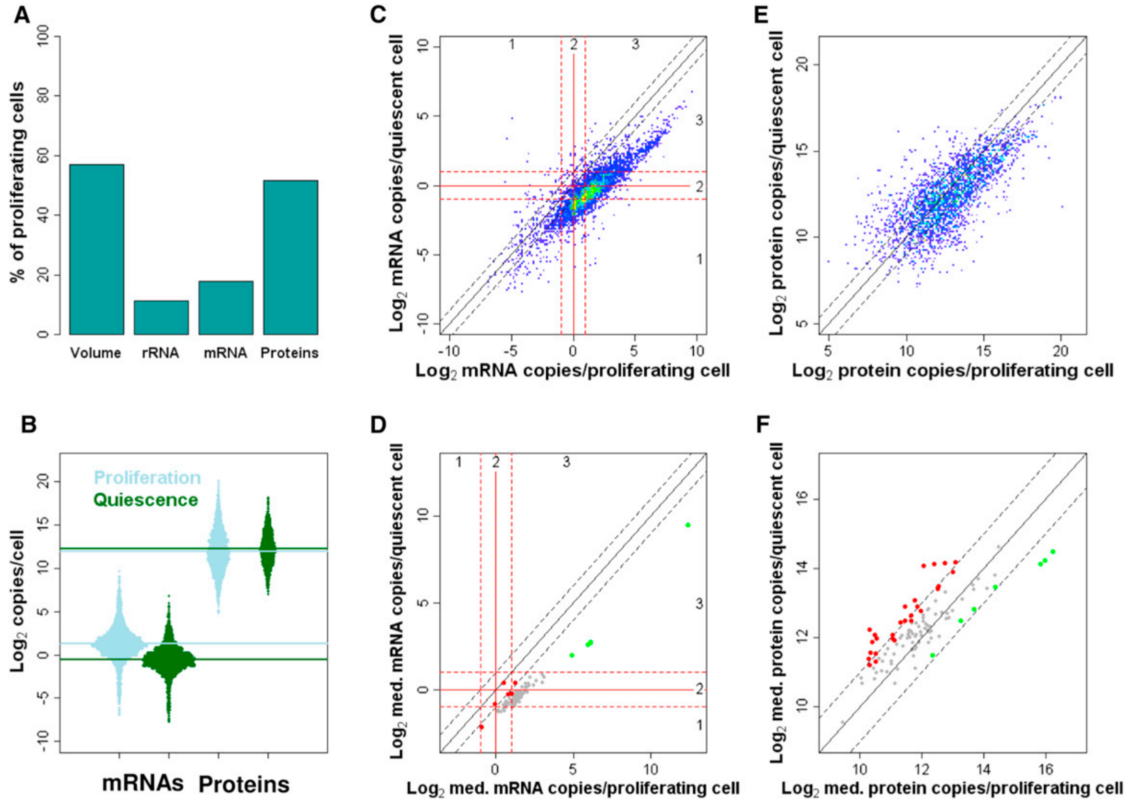

Figure 6. Transcriptomes and Proteomes in Proliferating versus Quiescent Cells

(A) Cell volume and rRNA, mRNA, and protein copy numbers in quiescent cells as percentage of corresponding values in proliferating cells.

(B) Distribution of mRNA (left) and protein (right) copies/cell during proliferation (blue) and quiescence (green), with median mRNA and protein abundance during proliferation and quiescence indicated by horizontal blue and green lines, respectively.

(C) mRNA abundance in quiescent versus proliferating cells. Red and black lines delimit expression zone 2 (0.5–2 mRNA copies/cell) and 2-fold expression changes, respectively.

(D) Median mRNA abundance of selected functional categories in quiescent versus proliferating cells. Red and black lines as in (C). Red and green dots indicate lowly and highly repressed categories, respectively (Table S14).

(E) Protein abundance in quiescent versus proliferating cells. Black diagonal lines delimit 2-fold expression changes.

(F) Median protein abundance of selected functional categories in quiescent versus proliferating cells. Black lines as in (E). Red and green dots indicate induced and repressed categories, respectively (Table S14).

copy numbers (Figure 6D). In conclusion, quiescence is characterized by a global reduction in mRNA numbers, but much less so in mRNA diversity. mRNAs involved in cell maintenance, such as adaptation to stress and nutrient limitation, become relatively more prevalent during quiescence, whereas those involved in translation become relatively less prevalent, although they remain highly abundant.

Proteome Does Not Shrink Globally but Is Remodeled during Quiescence

We detected a total of 31.2 million protein molecules/quiescent cell, representing 51.7% of the number measured in proliferating cells. Adjusting for the decreased volume of quiescent cells, however, protein numbers were only reduced by ~9.5%. Thus, the proteome largely scaled with volume, and, in stark contrast to the RNAs, quiescent cells maintained similar protein concentrations (Figures 6A and 6B). The median number of protein copies/quiescent cell was 4,851, which is actually higher than for proliferating cells. This apparent paradox is explained by a disproportionate reduction of the 10% most highly expressed proteins, involved in translation and growth, which account for 87.2% of all proteins lost in quiescent cells (Figures 6B and 6E). We detected 53.2% of all proteins during quiescence, ~13% less than during proliferation. The 897 proteins detected only in proliferating cells were enriched for mitochondrial translation and organization (Table S11). This finding suggests that quiescent cells have decreased oxidative metabolism, consistent with effects on mitochondrial translation and respiration on chronological lifespan (Pluskal et al., 2011). On the other hand, the 221 proteins detected only in quiescent cells were enriched for stress and nitrogen starvation functions.

The proteome was substantially remodeled during quiescence, with 47% of all proteins changing their copy numbers >2-fold (Figure 6E). Figure 6F illustrates this remodeling by comparing median protein copy numbers for selected functional categories between proliferating and quiescent cells. Several

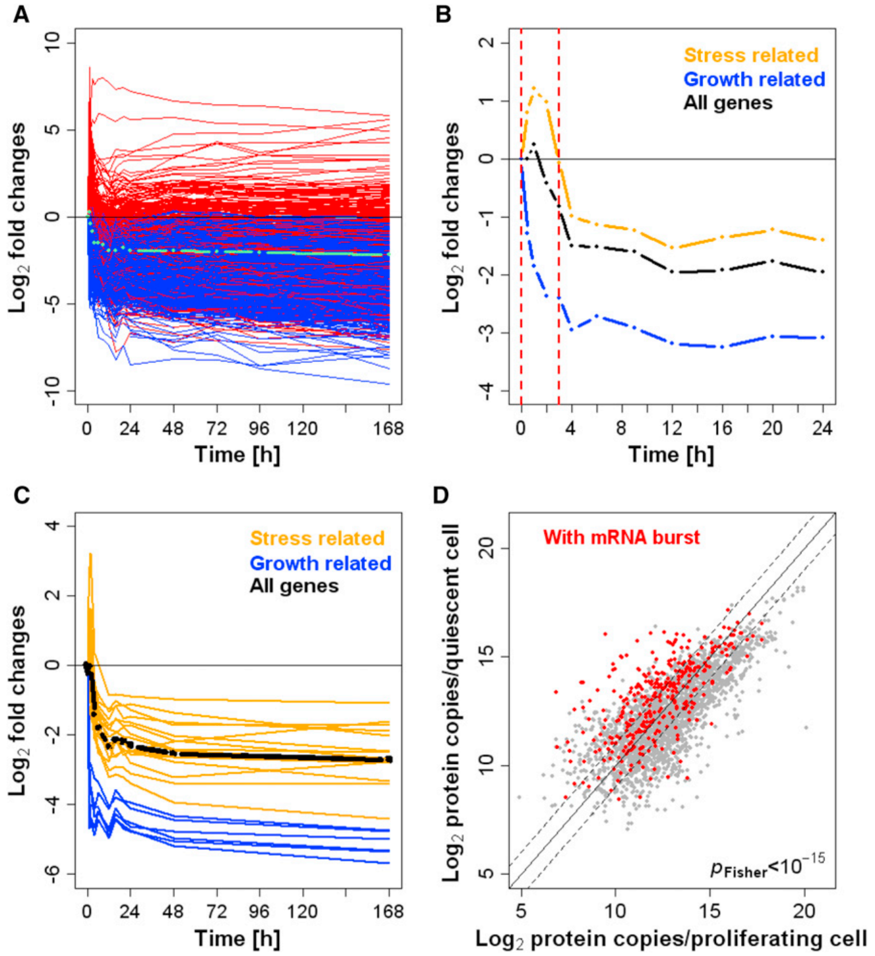

Figure 7. Regulatory Dynamics during Quiescence Entry

(A) Microarray time course to analyze changes in mRNA levels at 16 time points, before and 30 min to 7 days after nitrogen removal. Red profiles, mRNAs induced >1.5-fold within 3 hr after nitrogen removal; blue profiles, mRNAs repressed throughout time course. Data are normalized to 0 hr and corrected for total cellular RNA content.

(B) Average expression profiles of stress- and growth-related genes, and average expression changes of all genes.

(C) Absolute nCounter measurements of stress- and growth-related genes, and average profile for all 49 test genes.

(D) Protein abundance in quiescent versus proliferating cells. Lower right: significance of overlap between mRNAs induced >1.5-fold within 3 hr after nitrogen removal (red dots) and proteins induced >2-fold at 24 hr after nitrogen removal.

cellular maintenance functions, such as stress response, nitrogen starvation, DNA repair, vacuoles and cell wall, showed actually increased protein abundance in quiescence (Figure 6F; Table S14), in stark contrast to the global shrinking observed for mRNAs (Figure 6D). Categories with decreased protein abundance were related to translation and growth, similar to those strongly repressed at the mRNA level (Figure 6F). Notably, the top 50 most highly expressed proteins during proliferation were enriched for roles in glucose metabolism and translation, while during quiescence these proteins were only enriched for glucose metabolism (Table S11). This finding illustrates that quiescent cells remain metabolically active, while reducing the energetic costs of protein synthesis (Shimanuki et al., 2007). The differences in transcriptome and proteome regulation in quiescent cells resulted in a lowered correlation between mRNA and protein copies (R^2 = 0.36) compared to proliferating cells (R^2 = 0.55).

In conclusion, quiescent cells upregulate proteins implicated in a dormant lifestyle, while maintaining an abundant, yet strongly reduced, translational machinery. Together with the drastic reduction in overall mRNA abundance, this finding highlights the change in cellular physiology from a growth program for proliferation to a maintenance program for stress protection and long-term endurance. These two fundamental programs are implemented by balancing the expression of stress- versus growth-related genes, regulated by antagonistic signaling pathways such as the stress-activated protein kinase (SAPK) and target of rapamycin (TOR) (López-Maury et al., 2008).

Early mRNA Burst Sustains High Protein Numbers during Quiescence

We showed that 24 hr after nitrogen removal quiescent cells reached a state of a globally diminished transcriptome and a remodeled proteome. How do these cells manage to upregulate numerous proteins while downregulating most of the corresponding mRNAs (Figure 6)? We pursued this question by analyzing dynamic changes in mRNA levels at high temporal resolution. This time course experiment revealed that within 12 hr of nitrogen removal most mRNAs decreased whereas others transiently increased, followed by largely constant mRNA levels from 12–186 hr (Figure 7A). Although many stress-related genes were induced within 2 hr of nitrogen removal before becoming repressed, growth-related genes became immediately repressed (Figure 7B). We also measured absolute mRNA abundance for 49 genes from the same cell samples that reiterated the global data, excluding a normalization artifact (Figure 7C). Note that the average expression of all genes, and the absolute expression of the 49 test genes, decreased during the time course, with the stress-related genes showing a lower decrease and the growth-related mRNAs a higher decrease relative to all genes (Figures 7B and 7C). This pattern is also reflected in relative expression changes from microarray data (Mata and Bähler, 2006; Shimanuki et al., 2007). The absolute data presented here, however, expands and refines our understanding of this gene expression program, revealing that the upregulation

of stress-related genes is only transient, followed by a global repression of most genes.

Entry into quiescence thus consists of two phases: (1) a rapid adaptation where selected genes are induced, and (2) a global, but differential, repression of most genes. The burst in stress-related mRNAs could contribute to the proteome reshuffling observed at 24 hr. Indeed, a significant number of proteins with increased levels during quiescence corresponded to the transiently-induced mRNAs (Figure 7D). Thus, the early mRNA burst leads to a sustained increase of selected proteins, long after the corresponding mRNAs have decreased again. This mode of regulation depends on longer half-lives for proteins than for mRNAs, and it is plausible that proteins become further stabilized during quiescence. We conclude that cells, upon nitrogen removal, immediately repress the growth-related mRNAs while transiently inducing stress-related mRNAs, which in turn help to adjust the proteome for extended quiescence.

Conclusions

We comprehensively quantified the average numbers of RNAs and proteins in two fundamental cellular states of a eukaryotic model system. Besides providing a lasting resource for follow-up-studies, our data provide unique insight into cell regulation and function. Although mRNA and protein levels are well correlated overall, more strongly in proliferating than in quiescent cells, different mRNAs are ~10 to 60,000-fold less abundant than the corresponding proteins. This finding highlights the substantial amplification and regulation occurring during translation and protein turnover. Given that most RNAs are expressed at single-digit copy numbers, they are much more susceptible to stochastic events than proteins expressed at thousands of copies. Distinct expression zones for mRNAs and proteins reflect functional demands. Most mRNAs are expressed at ~1–10 copies/cell, whereas mRNAs present at well below 1 copy/cell are enriched for tightly repressed differentiation and regulatory genes that typically do not produce detectable proteins. This finding contrasts with data from bacteria where productive protein expression is achieved with such low mRNA concentrations (Taniguchi et al., 2010). Ultimately, population average measurements will need to be integrated with single-cell data to understand more complex cellular distributions of RNAs and proteins (Hebenstreit et al., 2011).

lncRNAs are generally present at well below 1 copy/cell, although ~200 lncRNAs, including ~40 nonpolyadenylated RNAs, are more robustly expressed at ~1–200 copies/cell and are thus prime candidates for functional analyses. However, the abundance of these lncRNAs is still much lower than for most proteins, suggesting functions with different biochemical characteristics. For instance, ~1 lncRNA copy/cell could be sufficient for roles in *cis*, where the RNA acts where it is transcribed, whereas higher expression levels could suggest roles in *trans*.

The transcriptome is larger in proliferating than in quiescent cells, reflecting the higher need for transcription during growth and division, and suggesting the existence of a global regulatory mechanism coordinating overall RNA abundance. In contrast, the proteome size is similar in proliferating and quiescent cells, after adjusting for differences in cell volume. However, the relative levels of numerous proteins show striking antagonistic changes in proliferating and quiescent cells, adapted for cellular growth or maintenance, respectively. Proteome remodeling during quiescence is enabled by a transient burst of stress-related mRNAs that leads to sustained high levels of the corresponding proteins. Protein concentrations are optimized to avoid molecular crowding for biochemical reactions (Dill et al., 2011). Constant protein concentrations during growth imply that protein numbers increase with cell volume, as do rRNA and ribosome numbers (Maclean, 1965) as well as mRNA numbers (Zhurinsky et al., 2010). Thus, the absolute cellular numbers of mRNA and protein molecules are not fixed by the genome but are globally tuned to cell volume and physiology. Genome-wide data on RNA and protein quantities are therefore vital to decipher the complex relationships linking genome regulation with cell physiology and growth, and to understand how different cells with identical genomes achieve the enormous diversity of functions. The findings reported here highlight elementary features of transcriptome and proteome regulation and provide a valuable platform to support future studies and quantitative biology.

EXPERIMENTAL PROCEDURES

Full methods are available in Extended Experimental Procedures.

Cell Cultures

Wild-type 972 h^- fission yeast cells were grown in Edinburgh minimal medium (EMM) at 32°C to mid-log phase; for quiescence experiments, such cells were shifted to EMM without nitrogen at 32°C and harvested at different times after nitrogen removal. Several cell pellets from the same cultures were frozen and used for RNA-seq, nCounter, and proteomics.

Quantitative Transcriptomics

RNA was extracted using the hot-phenol technique. Strand-specific RNA-seq libraries were prepared from total or poly(A)$^+$ RNA using an early version of the Illumina TruSeq Small RNA Sample Prep Kit. Sequencing scores were calculated as number of reads/kilobase. Scores derived from total RNA libraries were calibrated using absolute data acquired for 49 mRNAs, in whole cell extracts, on a nCounter instrument (NanoString), with external controls spiked in known quantities.

Quantitative Proteomics

Extracted proteins were enzymatically digested using trypsin, the peptides were separated into 12 fractions using an OFF-GEL Fractionator (Agilent), and analyzed on an Orbitrap Velos LC-MS platform (Thermo Scientific). Peptides were quantified and identified using the Progenesis LC-MS (Nonlinear Dynamics) and Mascot software, respectively. Absolute abundance for 39 proteins was determined using spiked-in heavy reference peptides to translate the summed MS-intensities of all peptides to copies/cell for all identified proteins.

Modeling of Cell-Cycle-Regulated mRNA Abundance

Periodic mRNA abundance was modeled for different cell-cycle phases using (1) average mRNA copies/cell in asynchronous cultures, (2) fraction of cell cycle with mRNA peak expression, and (3) amplitude of periodic mRNA regulation.

Quiescence Entry Time Course

RNA was extracted using the hot-phenol technique. Labeled cDNA of each sample was hybridized against a pool of all samples on a custom-designed

Agilent microarray. Absolute data for 49 test genes were acquired from the same cell samples using nCounter as described above.

ACCESSION NUMBERS

The ArrayExpress accession numbers for the RNA-seq and microarray data reported in this paper are MTAB-1154, and E-TABM-1075, respectively. The LC-MS data are available from ProteomeCommons.org Tranche using these hashes:

Proliferating cells OGE-fractions:
jsVcj9T6yAfpS80nYRueuobP0iOdpQzk/IgyYCYf+EZpj6fAE7dx9JoKvkhCfmQ
J5d5NuGLfriFt YEKJcB4rw+egFFcAAAAAAAACAw==

Quiescent cells OGE-fractions:
1gNlqHIUVaHU8S8yqpR8tvKvq6m0jySALyTlBxte1YvIo/N8IPXlMsgRKu2ZTg
sdPHPG0Z0jrrWtiDJ1CEXSb/fQY0kAAAAAAAACHg==

Unfractionated samples:
rSK2uX3cA5h/YrSal0U3y6Y0VneHG7p3J6fdXWAeLYC6HbnIejsgoKTgUNZL
We2Q+GRNQcc+xogxIG723ayC88ONM1cAAAAAAAACIg==

SUPPLEMENTAL INFORMATION

Supplemental information includes Extended Experimental Procedures, seven figures, and 18 tables and can be found with this article online at http://dx.doi.org/10.1016/j.cell.2012.09.019.

ACKNOWLEDGMENTS

We thank F. Bachand, D. Bitton, L. Foukas, A. Lock, D. Lovell, J. Mata, and V. Wood for comments on the manuscript, P. Gardner for advice on lncRNA sequences, and P. Oliveri for help with nCounter. This research was funded by PhenOxiGEn (an EU FP7 research project), a BBSRC Research Grant BB/I012451/1, and a Wellcome Trust Senior Investigator Award to J.B., and by SystemsX.ch and an ERC Advanced Grant to R.A.

Received: February 10, 2012
Revised: June 11, 2012
Accepted: July 26, 2012
Published: October 25, 2012

REFERENCES

Atkinson, S.R., Marguerat, S., and Bähler, J. (2012). Exploring long non-coding RNAs through sequencing. Semin. Cell Dev. Biol. *23*, 200–205.

Baer, B.W., and Kornberg, R.D. (1983). The protein responsible for the repeating structure of cytoplasmic poly(A)-ribonucleoprotein. J. Cell Biol. *96*, 717–721.

Beck, M., Claassen, M., and Aebersold, R. (2011). Comprehensive proteomics. Curr. Opin. Biotechnol. *22*, 3–8.

Bhavsar, R.B., Makley, L.N., and Tsonis, P.A. (2010). The other lives of ribosomal proteins. Hum. Genomics *4*, 327–344.

Chen, D., Toone, W.M., Mata, J., Lyne, R., Burns, G., Kivinen, K., Brazma, A., Jones, N., and Bähler, J. (2003). Global transcriptional responses of fission yeast to environmental stress. Mol. Biol. Cell *14*, 214–229.

Coller, H.A. (2011). Cell biology. The essence of quiescence. Science *334*, 1074–1075.

Cook, P.R. (2010). A model for all genomes: the role of transcription factories. J. Mol. Biol. *395*, 1–10.

Cox, J., and Mann, M. (2011). Quantitative, high-resolution proteomics for data-driven systems biology. Annu. Rev. Biochem. *80*, 273–299.

de Almeida, S.F., and Carmo-Fonseca, M. (2012). Design principles of interconnections between chromatin and pre-mRNA splicing. Trends Biochem. Sci. *37*, 248–253.

Dill, K.A., Ghosh, K., and Schmit, J.D. (2011). Physical limits of cells and proteomes. Proc. Natl. Acad. Sci. USA *108*, 17876–17882.

Dobbyn, H.C., and O'Keefe, R.T. (2004). Analysis of Snu13p mutations reveals differential interactions with the U4 snRNA and U3 snoRNA. RNA *10*, 308–320.

Gardner, P.P., Daub, J., Tate, J., Moore, B.L., Osuch, I.H., Griffiths-Jones, S., Finn, R.D., Nawrocki, E.P., Kolbe, D.L., Eddy, S.R., and Bateman, A. (2011). Rfam: Wikipedia, clans and the "decimal" release. Nucleic Acids Res. *39*(Database issue), D141–D145.

Grewal, S.I. (2010). RNAi-dependent formation of heterochromatin and its diverse functions. Curr. Opin. Genet. Dev. *20*, 134–141.

Harigaya, Y., and Yamamoto, M. (2007). Molecular mechanisms underlying the mitosis-meiosis decision. Chromosome Res. *15*, 523–537.

Hebenstreit, D., Fang, M., Gu, M., Charoensawan, V., van Oudenaarden, A., and Teichmann, S.A. (2011). RNA sequencing reveals two major classes of gene expression levels in metazoan cells. Mol. Syst. Biol. *7*, 497.

Hogan, D.J., Riordan, D.P., Gerber, A.P., Herschlag, D., and Brown, P.O. (2008). Diverse RNA-binding proteins interact with functionally related sets of RNAs, suggesting an extensive regulatory system. PLoS Biol. *6*, e255.

Holstege, F.C., Jennings, E.G., Wyrick, J.J., Lee, T.I., Hengartner, C.J., Green, M.R., Golub, T.R., Lander, E.S., and Young, R.A. (1998). Dissecting the regulatory circuitry of a eukaryotic genome. Cell *95*, 717–728.

Kim, D.U., Hayles, J., Kim, D., Wood, V., Park, H.O., Won, M., Yoo, H.S., Duhig, T., Nam, M., Palmer, G., et al. (2010). Analysis of a genome-wide set of gene deletions in the fission yeast Schizosaccharomyces pombe. Nat. Biotechnol. *28*, 617–623.

Lackner, D.H., Beilharz, T.H., Marguerat, S., Mata, J., Watt, S., Schubert, F., Preiss, T., and Bähler, J. (2007). A network of multiple regulatory layers shapes gene expression in fission yeast. Mol. Cell *26*, 145–155.

Larson, D.R., Singer, R.H., and Zenklusen, D. (2009). A single molecule view of gene expression. Trends Cell Biol. *19*, 630–637.

Lemay, J.F., D'Amours, A., Lemieux, C., Lackner, D.H., St-Sauveur, V.G., Bähler, J., and Bachand, F. (2010). The nuclear poly(A)-binding protein interacts with the exosome to promote synthesis of noncoding small nucleolar RNAs. Mol. Cell *37*, 34–45.

Li, L., and Clevers, H. (2010). Coexistence of quiescent and active adult stem cells in mammals. Science *327*, 542–545.

López-Maury, L., Marguerat, S., and Bähler, J. (2008). Tuning gene expression to changing environments: from rapid responses to evolutionary adaptation. Nat. Rev. Genet. *9*, 583–593.

Lovell, D., Muller, W., Taylor, J., Zwart, A., and Helliwell, C. (2011). Proportion, percentages, PPM: do the molecular biosciences treat compositional data right? In Compositional Data Analysis: Theory and Applications, V. Pawlowsky-Glahn, A. Buccianti, D. Lovell, W. Müller, J. Taylor, A. Zwart, and C. Helliwell, eds. (New York: John Wiley & Sons), pp. 193–206.

Maclean, N. (1965). Ribosome numbers in a fission yeast. Nature *207*, 322–323.

Maier, T., Schmidt, A., Güell, M., Kühner, S., Gavin, A.C., Aebersold, R., and Serrano, L. (2011). Quantification of mRNA and protein and integration with protein turnover in a bacterium. Mol. Syst. Biol. *7*, 511.

Marguerat, S., Jensen, T.S., de Lichtenberg, U., Wilhelm, B.T., Jensen, L.J., and Bähler, J. (2006). The more the merrier: comparative analysis of microarray studies on cell cycle-regulated genes in fission yeast. Yeast *23*, 261–277.

Mata, J., and Bähler, J. (2006). Global roles of Ste11p, cell type, and pheromone in the control of gene expression during early sexual differentiation in fission yeast. Proc. Natl. Acad. Sci. USA *103*, 15517–15522.

Mata, J., Lyne, R., Burns, G., and Bähler, J. (2002). The transcriptional program of meiosis and sporulation in fission yeast. Nat. Genet. *32*, 143–147.

Mata, J., Wilbrey, A., and Bähler, J. (2007). Transcriptional regulatory network for sexual differentiation in fission yeast. Genome Biol. *8*, R217.

McPheeters, D.S., Cremona, N., Sunder, S., Chen, H.M., Averbeck, N., Leatherwood, J., and Wise, J.A. (2009). A complex gene regulatory mechanism that

operates at the nexus of multiple RNA processing decisions. Nat. Struct. Mol. Biol. *16*, 255–264.

Miura, F., Kawaguchi, N., Yoshida, M., Uematsu, C., Kito, K., Sakaki, Y., and Ito, T. (2008). Absolute quantification of the budding yeast transcriptome by means of competitive PCR between genomic and complementary DNAs. BMC Genomics *9*, 574.

Nagaraj, N., Wisniewski, J.R., Geiger, T., Cox, J., Kircher, M., Kelso, J., Pääbo, S., and Mann, M. (2011). Deep proteome and transcriptome mapping of a human cancer cell line. Mol. Syst. Biol. *7*, 548.

Ng, S.S., Anderson, M., White, S., and McInerny, C.J. (2001). mik1(+) G1-S transcription regulates mitotic entry in fission yeast. FEBS Lett. *503*, 131–134.

Ozsolak, F., and Milos, P.M. (2011). RNA sequencing: advances, challenges and opportunities. Nat. Rev. Genet. *12*, 87–98.

Pancaldi, V., Schubert, F., and Bähler, J. (2010). Meta-analysis of genome regulation and expression variability across hundreds of environmental and genetic perturbations in fission yeast. Mol. Biosyst. *6*, 543–552.

Pluskal, T., Hayashi, T., Saitoh, S., Fujisawa, A., and Yanagida, M. (2011). Specific biomarkers for stochastic division patterns and starvation-induced quiescence under limited glucose levels in fission yeast. FEBS J. *278*, 1299–1315.

Rhind, N., Chen, Z., Yassour, M., Thompson, D.A., Haas, B.J., Habib, N., Wapinski, I., Roy, S., Lin, M.F., Heiman, D.I., et al. (2011). Comparative functional genomics of the fission yeasts. Science *332*, 930–936.

Rustici, G., Mata, J., Kivinen, K., Lió, P., Penkett, C.J., Burns, G., Hayles, J., Brazma, A., Nurse, P., and Bähler, J. (2004). Periodic gene expression program of the fission yeast cell cycle. Nat. Genet. *36*, 809–817.

Schmidt, A., Beck, M., Malmström, J., Lam, H., Claassen, M., Campbell, D., and Aebersold, R. (2011). Absolute quantification of microbial proteomes at different states by directed mass spectrometry. Mol. Syst. Biol. *7*, 510.

Schmidt, M.W., Houseman, A., Ivanov, A.R., and Wolf, D.A. (2007). Comparative proteomic and transcriptomic profiling of the fission yeast Schizosaccharomyces pombe. Mol. Syst. Biol. *3*, 79.

Schwanhäusser, B., Busse, D., Li, N., Dittmar, G., Schuchhardt, J., Wolf, J., Chen, W., and Selbach, M. (2011). Global quantification of mammalian gene expression control. Nature *473*, 337–342.

Shimanuki, M., Chung, S.Y., Chikashige, Y., Kawasaki, Y., Uehara, L., Tsutsumi, C., Hatanaka, M., Hiraoka, Y., Nagao, K., and Yanagida, M. (2007). Two-step, extensive alterations in the transcriptome from G0 arrest to cell division in Schizosaccharomyces pombe. Genes Cells *12*, 677–692.

Strässer, K., and Hurt, E. (2001). Splicing factor Sub2p is required for nuclear mRNA export through its interaction with Yra1p. Nature *413*, 648–652.

Taniguchi, Y., Choi, P.J., Li, G.W., Chen, H., Babu, M., Hearn, J., Emili, A., and Xie, X.S. (2010). Quantifying E. coli proteome and transcriptome with single-molecule sensitivity in single cells. Science *329*, 533–538.

Vogel, C., and Marcotte, E.M. (2012). Insights into the regulation of protein abundance from proteomic and transcriptomic analyses. Nat. Rev. Genet. *13*, 227–232.

Wilhelm, B.T., Marguerat, S., Watt, S., Schubert, F., Wood, V., Goodhead, I., Penkett, C.J., Rogers, J., and Bähler, J. (2008). Dynamic repertoire of a eukaryotic transcriptome surveyed at single-nucleotide resolution. Nature *453*, 1239–1243.

Wood, V., Harris, M.A., McDowall, M.D., Rutherford, K., Vaughan, B.W., Staines, D.M., Aslett, M., Lock, A., Bähler, J., Kersey, P.J., and Oliver, S.G. (2012). PomBase: a comprehensive online resource for fission yeast. Nucleic Acids Res. *40*(Database issue), D695–D699.

Wu, J.Q., and Pollard, T.D. (2005). Counting cytokinesis proteins globally and locally in fission yeast. Science *310*, 310–314.

Yanagida, M. (2009). Cellular quiescence: are controlling genes conserved? Trends Cell Biol. *19*, 705–715.

Zenklusen, D., Larson, D.R., and Singer, R.H. (2008). Single-RNA counting reveals alternative modes of gene expression in yeast. Nat. Struct. Mol. Biol. *15*, 1263–1271.

Zhurinsky, J., Leonhard, K., Watt, S., Marguerat, S., Bähler, J., and Nurse, P. (2010). A coordinated global control over cellular transcription. Curr. Biol. *20*, 2010–2015.

Zofall, M., Yamanaka, S., Reyes-Turcu, F.E., Zhang, K., Rubin, C., and Grewal, S.I. (2012). RNA elimination machinery targeting meiotic mRNAs promotes facultative heterochromatin formation. Science *335*, 96–100.

Supplemental Information

EXTENDED EXPERIMENTAL PROCEDURES

Cell Culture Conditions

Three cultures of wild-type 972 h- cells were grown in Edinburgh minimal medium (EMM) at 32°C to 7.0-7.4 x10^6 cells/ml (called MM1 to MM3). Cells were harvested, and several pellets from same cultures were frozen for RNA-seq, NanoString, and proteomics experiments. The remaining of the cultures were then washed twice in EMM without nitrogen source (NH_4Cl), and cultured for 24h in EMM without nitrogen at 32°C; multiple pellets were then harvested as above (called MN1-MN3). For the entry into quiescence timecourse experiments, cells were treated as above and multiple pellets were frozen before and at 15 time points after nitrogen removal from the medium (30min, 1h, 2h, 3h, 4h, 6h, 9h, 12h, 16h, 20h, 24h, 48h, 3 days, 5 days, and 7 days).

Measurement of Relative RNA Levels

Relative RNA abundance of two cell cultures grown in EMM (MM1 and MM2) and two cultures incubated in absence of nitrogen for 24h (MN1, MN2) were obtained by RNA-seq. For each sample, we created two libraries: one from total RNA (no poly(A)$^+$ selection or rRNA depletion step), and one from poly(dT)-enriched RNA. Strand-specific RNA-seq libraries were prepared using an unreleased early version of the Illumina TruSeq Small RNA Sample Prep Kit. Briefly, total RNA was isolated by hot-phenol extraction, and RNA quality was assessed on a Bioanalyzer instrument (Agilent). For the poly(dT)-enriched libraries, 10 μg of total RNA was used as starting material, and poly(A)$^+$ RNA was enriched by two rounds of poly(dT) Sera-Mag magnetic bead purification. For the total RNA libraries, 100ng of total RNA was used as starting material without any prior treatment. RNA was fragmented to an average size of ~200nt. Fragmented RNA was 3′ de-phosphorylated with Antartic phosphatase and 5′ phosphorylated with polynucleotide kinase; this treatment prepares RNA fragments for subsequent ligation of Illumina RNA adaptors to their 5′ and 3′ ends using a 3′ RNA ligase and a T4 RNA ligase, respectively. First-strand cDNA was produced using a primer specific for the Illumina 3′ adaptor. The library was amplified with 15 PCR cycles using primers specific for the Illumina adaptors and purified using SPRI-beads (Agencourt, Beckman Coulter). Library size distributions and concentrations were determined on a Bioanalyzer (Agilent), RNA-seq libraries were sequenced on an Illumina Genome Analyzer IIx (poly(dT) enriched libraries), or an Illumina HiSeq 2000 instrument (total RNA libraries). Between 238.6 and 288.8 million reads were obtained for the total RNA libraries, and between 33.3 and 41.8 million reads for the (poly(dT) enriched libraries (Table S1).

Reads were aligned to the fission yeast genome (Wood et al., 2002) with the exonerate software (Slater and Birney, 2005), and reads matching to multiple locations in the genome were assigned at random to only one of these locations. Reads containing up to 5 mismatches (not clustered at read ends) were kept for further analysis (“Genomic set”). The remaining sequencing reads were then mapped against fission yeast spliced CDS (Wood et al., 2002) and filtered as above (“Spliced set”). Pools of both sets of reads were used for further analyses. We obtained between 172.7 and 201.4 million mappable reads for the total RNA libraries and between 28.2 and 33.3 mappable reads for the poly(dT) enriched libraries (Table S1). To compare data sets with similar mRNA coverage, 5.5 million reads were randomly selected from the poly(dT)-enriched data and used for further analysis. The correlation between expression levels in RPKM computed from the poly(dT) data before and after sub-sampling was very high ($R_{Pearson} > 0.99$). Figure S1A shows the distribution of the number of reads per annotated features from different RNA categories in both total and poly(dT) enriched MM1 libraries. Figure S1A indicates that poly(dT) enrichment has minor global effects on read numbers for ncRNA and long terminal repeats (LTR) derived transcripts, while reads matching rRNA, tRNA, snRNA, snoRNA, and mitochondria-encoded RNA are depleted by the poly(dT) treatment. Table S1 contains the distribution of reads in these categories for all 8 data sets and confirms these observations. In all data sets, > 93% of mRNAs were hit by > 10 reads and > 74% were hit > 100 times. Finally, in all 8 data sets, at least 90% of all exons had coverage of 50% and above.

Relative sequencing expression scores were calculated for spliced transcripts, introns, and regions antisense to open reading frames, using the genome annotation available in GeneDB (http://old.genedb.org/, now PomBase http://www.pombase.org/) on 9th May 2011. The number of reads mapping to each feature was divided by the length of the feature in kilobase [RPK]. Sequencing scores were based on annotated exon boundaries excluding UTRs to avoid variability due to alternative UTR usage and to increase accuracy (Ramsköld et al., 2009). The scores were corrected for inaccuracies arising for possible uneven mappability across the genome and biases introduced during score calculation. Briefly, simulated reads covering the fission yeast genome were produced using the ‘maq simulate’ function of the Maq package (Li et al., 2008). This data set was then processed exactly as the RNA-seq data. Relative sequencing expression scores derived from this simulated data set were median-centered and used as correction factors for the RNA-seq data. Figure S1B shows the distribution of simulated sequencing scores as a function of feature size, indicating that shorter features are characterized by increased biases, although overall correction factors remained close to unity. Note that three RNA families (tRNAs, snRNAs, snoRNAs), despite being present at native levels in total RNA samples (Figure S1A), are not only very short but also highly modified, implying additional, uncontrolled biases that might affect the accuracy of their quantification. Corrected scores spanned 7 and 5 orders of magnitude, for total and poly(dT) enriched libraries, respectively, and showed strong correlation between biological replicates ($r_{pearson} > 0.98$ in each data set; Table S2).

In order to identify and flag genes in regions of the genome more difficult to sequence, we have compared read counts in RPKM for each gene, calculated from a set of simulated reads based on the fission yeast reference sequence, with read counts for each gene derived from DNA sequencing from the same strain. Read counts were median-centered, and a “DNA over simulated” ratio was

calculated. Genes with ratios largely below 1 were likely derived from regions difficult to sequence and were enriched for tRNAs and lncRNAs. Genes with ratios largely above 1 were mainly rRNAs, mitochondrially encoded, or located in subtelomeric regions. This pattern reflects the lack of information on the exact number of duplicated rRNA genes in the fission yeast genome, the presence of multiple mitochondrial genomes per cell, and the probable gene duplications occurring in subtelomeric regions. The ratios from this analysis are provided in Table S4 in a column called "sequencibility."

Genome-wide Measurement of Absolute RNA Copies/Cell

To calibrate relative RNA-seq scores and to obtain absolute RNA abundance for the entire transcriptome, we first measured the absolute copy numbers of 49 mRNAs using an nCounter instrument (NanoString) (Table S3). This calibration set was designed such that sequencing scores of > 90% of all mRNA were within the expression interval covered by the 49 calibration mRNAs. The NanoString technology uses two sets of probes: one is coupled to fluorescent barcodes and permits to tag individual mRNAs, while the other one is used to immobilise barcode-bound mRNAs to a glass slide on which individual barcoded mRNAs are counted using an optical camera. This approach is digital and highly quantitative, does not require any enzymatic reactions, and is performed on whole-cell extracts, bypassing the need of RNA purification.

Cells from the same cultures used for RNA-seq library preparation were resuspended in RLT buffer (QIAGEN) and counted by an automated cell counter (Coulter) and haemocytometer. Cells were broken using a FastPrep instrument (MP Biomedicals) on setting 6.5 for 2 × 1min. Total cell lysis was > 90% for all samples. Cell extract volumes corresponding to 5×10^4 cells were used for mRNA quantification on nCounter (NanoString) using the manufacturer's instructions for samples MM1, MM2, MN1, and MN2. Data were extracted and normalized according to the manufacturer's instructions. Three technical repeats for each sample were obtained from two independent nCounter runs. To convert nCounter counts to copies/cell, 13 external controls were added at known concentrations to the cell extracts. Six external controls were provided by the manufacturer as part of the NanoString mastermix (Figures S1C and S1D, gray circles), while 7 external controls were bought separately (AM1780, Ambion) and were added directly to the cell extracts (Figures S1C and S1D, gray triangles). Spike concentrations were designed to cover the expression range of the 49 calibration mRNAs and were confirmed on a Bioanalyzer (Agilent) before being added to the extracts. The average of 12 measurements/slide for each external control was used for normalization. In both runs some external controls appeared outside the dynamic range of the nCounter. To avoid biases, we discarded extreme data points while making sure that the calibration curve would be supported experimentally throughout the concentration spectrum of the 49 mRNAs used for calibration of the RNA-seq data (quantity of lowest and highest expressed genes from the 49 mRNA set are marked by blue lines on Figures S1C and S1D). Correlation between absolute spike copies and nCounter counts was high in both runs ($r_{pearson} > 0.98$). The copies/cell for the calibration set were then calculated using linear regression of the log2 transformed external control values. Figures S1E–S1H shows the distribution of the coefficient of variation (σ/μ) of the measured concentrations in copies/cell between the 3 technical repeats for each sample. The absolute measurements of calibration mRNAs from the 3 technical repeats were averaged (first within and then between runs) and used for calibration of the RNA-seq data.

Corrected relative sequencing scores from RNA-seq libraries derived from total RNA were calibrated using using linear regression of the log2 transformed absolute expression values (copies/cell) obtained for the 49 calibration mRNAs (see above, Table S3). Figures S1K and S1M show the strong correlation between absolute copies/cell and relative sequencing scores for the MM1 and MN1 samples, respectively. Figures S1L and S1N show the distribution of measurement error using a linear model described here (Schmidt et al., 2011) and indicates that the mean measurement error is 2-fold or lower. The correlation of absolute values between biological replicates was high ($r_{pearson} > 0.98$, Figures S1I and S1J). For that reason, the average of the biological repeats was used for all downstream analysis.

Entry into Quiescence Time Course Experiments

Cells were harvested before and at 15 time points after nitrogen removal from the medium (see above). For microarray analysis, total RNA was extracted by the hot-phenol method (Lyne et al., 2003), and purified once using the RNeasy purification kit, and twice using RNeasy mini-elute kits (QIAGEN). These extended washes minimized the carryover of contaminants present in quiescent cells which can inhibit cDNA labeling (Bähler lab observation). Total RNA was labeled using a SuperScript Plus Direct cDNA Labeling System (Invitrogen), using a reduced amount of labeled nucleotides to minimize dye-bias (Juan Mata, personal communication). Labeled samples were hybridized against a pool of all samples on a custom-designed Agilent platform containing all coding and non-coding features present in GeneDB (http://old.genedb.org/, now PomBase http://www.pombase.org/; Wood et al., 2012) in April 2011. Microarrays were hybridized and washed according to manufacturer's instructions, and scanned on an Axon 4000B scanner using the GenePix 6.0 software. Background was subtracted, and arrays were normalized using the loess-based method available in the bioconductor package limma (http://www.bioconductor.org). A pooled reference was included for technical reasons, and ratios for all genes were divided by their ratios at time point zero before nitrogen removal to obtain biologically meaningful expression ratios. To take into account the global reduction of total RNA concentration per cell during entry into quiescence (see main text), expression ratios for each time point were multiplied by the fraction of total RNA/cell present at the given time point compared to cells before nitrogen removal (Table S15).

For time points 0, 30min, 1h, 2h, 4h, 6h, 12h, 16h, 20h, 24h, 48h and 7 days, we performed nCounter measurements of absolute abundance for 49 mRNAs as described above using cells from the same cultures as for microarrays (Table S16).

Sample Preparation for Proteome Analysis

Around 10^8 cells of each of the 6 samples (MM1-3 and MN1-3) were washed twice with PBS buffer, harvested by centrifugation at 2,000 g, and resuspended in 100µl lysis buffer (100 mM ammoniumbicarbonate, 8M urea, 0.1% RapiGest) containing 100µl of glass beads. The cells were disrupted first by strong vortexing for 3 × 30 s using a *FastPrep*® FP120 Cell Disrupter (Thermo Fisher Scientific) followed by sonication for 3 × 20 s. A small aliquot of the supernatant was taken to determine the protein concentration of each sample using a BCA assay (Thermo Fisher Scientific). Proteins obtained from the different samples were reduced with 5mM TCEP for 60min at 37°C and alkylated with 10mM iodoacetamide for 30min in the dark at 25°C. After quenching the reaction with 12 mM N-acetyl-cysteine, the proteins were proteolyzed for 4h at 37°C using sequencing-grade Lys-C (Wako Chemicals) at 1/200 w/w. Then, the samples were diluted with 100mM ammoniumbicarbonate buffer to a final urea concentration of 1.5M and further digested by incubation with sequencing-grade modified trypsin (1/50, w/w; Promega, Madison, Wisconsin) over night at 37°C. The samples were acidified with 2M HCl to a final concentration of 50mM, incubated for 15min at 37°C and the cleaved detergent removed by centrifugation at 10,000 g for 15min. Subsequently, an aliquot of the 39 AQUA peptide mix (see Table S5 for details) containing 200/20 fmol of heavy peptides per 1µg of endogenous proteins was spiked in each sample. All peptide samples were then desalted by C18 reversed-phase spin columns according to the manufacturer's instructions (Macrospin, Harvard Apparatus), dried under vacuum and stored at −80°C until further use. For direct LC-MS analysis, samples were solubilized in solvent A (98% water, 2% acetonitrile, 0.15% formic acid) at a concentration of 0.5 µg/µl and 2 µl were injected per run.

Off-Gel Electrophoresis

Two peptide mixtures for each growth condition (MM and MN), containing equal aliquots of 100 µg dried peptides of the 3 biological replicates (MM1-3 and MN1-3), respectively, were resolubilized in 1800 µl Off-Gel electrophoresis buffer according to the manufacturer's instructions (3100 OFFGEL Fractionator, Agilent Technologies). Both peptide samples were then separated on a 12cm pH 3-10 IPG strip (GE Healthcare), respectively, using a protocol of 1h rehydration at maximum 500V, 50 µA and 200 mW. Peptides were separated at maximum 8000V, 100 µA and 300mW until 20kVh was reached. Subsequently, each of the 12 peptide fractions was desalted using C18 reversed-phase columns according to the manufacturer's instructions (Microspin, Harvard Apparatus), dried under vacuum and subjected to LC-MS/MS analysis.

LC-MS/MS Analysis

The setup of the µRPLC-MS system was as described previously (Schmidt et al., 2011; Beck et al., 2011) with some modifications. The hybrid Orbitrap-Velos mass spectrometer was interfaced to a nanoelectrospray ion source coupled online to an Easy-nLC system (all ThermoScientific). 1µg of peptides were separated on a RP-LC column (75 µm x 20 cm) packed in-house with C18 resin (Magic C18 AQ 3 µm; Michrom BioResources) using a linear gradient from 95% solvent A (98% water, 2% acetonitrile, 0.15% formic acid) and 5% solvent B (98% acetonitrile, 2% water, 0.15% formic acid) to 30% solvent B over 90min for OGE-fractions and 120 min for unfractionated samples at a flow rate of 0.3µl/min. Each survey scan acquired in the Orbitrap at 60,000 FWHM was followed by 10 MS/MS scans of the most intense and 2 additional MS/MS scans of the spiked in AQUA peptide pair precursor ions in the linear ion trap using a mass inclusion list. Preview mode was enabled and dynamic exclusion was set for 60 s. Charge state screening was employed to select for ions with at least two charges and rejecting ions with undetermined charge state. The normalized collision energy was set to 32%, and one microscan was acquired for each spectrum.

Protein Identification and Quantification

The acquired raw-files were imported into the Progenesis LC-MS software (v3.0, Nonlinear Dynamics Limited), which was used to extract peptide precursor ion intensities across all samples applying the default parameters. The generated mgf-files were searched using MASCOT against a decoy database (consisting of forward and reverse protein sequences; Elias and Gygi, 2007) of the predicted *S. pombe* proteome (ftp://ftp.sanger.ac.uk/pub/yeast/pombe/Protein_data/pompep). The database consists of 5143 *S. pombe* proteins as well as known contaminants such as porcine trypsin, human keratins and high abundant bovine serum proteins (Non-Redundant Protein Database, National Cancer Institute Advanced Biomedical Computing Center, 2004, ftp://ftp.ncifcrf.gov/pub/nonredundant), resulting in a total of 10,584 protein sequences. The search criteria were set as follows: full tryptic specificity was required (cleavage after lysine or arginine residues, unless followed by proline); 2 missed cleavages were allowed; carbamidomethylation (C) was set as fixed modification; oxidation (M) was applied as variable modifications; mass tolerance of 10 ppm (precursor) and 0.6Da (fragments). The database search results were filtered using the ion score to set the false discovery rate (FDR) to 1% on the peptide and protein level, respectively, based on the number of reverse protein sequence hits in the data sets. The quantitative data obtained were further normalized and statistically analyzed according to Brusniak et al. (2008).

Absolute Protein Quantification

Absolute quantification was carried out according to (Schmidt et al., 2011). In brief, the raw files were converted to mzXML file format, the database searched using XTandem/PeptideProphet followed by isotope ratio calculation by the Xpress software tool. All software tools are part of the trans-proteomics pipeline (Deutsch et al., 2010). The median peptide ratios were used to determine the endogenous protein levels in the individual samples. Based on the number of disrupted cells counted in triplicates for each sample before and after cell lysis, absolute abundances for the selected proteins (in copies/cell) could be calculated across all samples

(Tables S7 and S8). These were aligned with the summed protein intensities as provided by the Progenesis LC-MS software (v3.0, Nonlinear Dynamics Limited) divided by the number of expected tryptic peptides. The thus generated models were applied to estimate absolute protein levels for all quantified proteins in the individual samples (see Tables S5–S9). To assess the technical as well as technical & biological variability of our label-free quantification approach, we performed duplicate LC-MS analyses of each biological triplicate sample and determined the expected mean, average lower and upper endpoint of 95% confidence interval (L95 and U95) expression variations as a function of the number of available peptides per protein (Figures S3A–S3D). Additionally, hierarchical clustering of protein concentrations confirmed the low technical and biological variations in our quantitative data set (Figure S3E).

For further evaluation of our absolute protein quantities, we compared abundances of proteins being present in five stable protein complexes with an expected stoichiometry of 1:1. In all cases, the protein abundances matched the expected values within calculated error rates for both growth conditions (Table S18), providing further evidence that the estimated protein levels are accurate.

Measurement of Cell Size and Volume

Cells were fixed for 15 min in culture medium supplemented with ~4% formaldehyde, and washed 3 times in PBS. Cell pictures were obtained on a Zeiss Axioskop microscope with plan-Apochromat 63x 1.4 NA oil immersion objective, and cell lengths and widths were measured by hand using the ImageJ software (http://rsbweb.nih.gov/ij/) and the plugin ObjectJ (http://simon.bio.uva.nl/objectj/). Cell volume was calculated as in (Shimanuki et al., 2007):

$$V = \frac{\pi D^2 \left(L - \frac{D}{3}\right)}{4}$$

where V is the cell volume, D the cell diameter and L the cell length.

Analysis of Cell-Cycle-Regulated Genes

First, the total number of molecules of a given mRNA in an asynchronous culture can be expressed as relation (1), assuming two different cell types with distinct levels of a transcript *i* and the absence of detectable intermediate states:

$$C^i_{tot} \times N_{tot} = \left(C^i_{bas} \times N_{bas}\right) + \left(C^i_{peak} \times N_{peak}\right) \quad (1)$$

Where, C^i_{tot} represents the average number of mRNA copies/cell in the culture, C^i_{bas} the average number of mRNA copies/cell in the phase(s) of the cell-cycle were expression is low or ‘basal’, and C^i_{peak} the mRNA copies/cell in the phase of the cell-cycle were gene expression peaks. $N_{tot}, N_{bas}, N_{peak}$ are the numbers of cells in the culture, in the ‘basal’ phase(s) of the cycle and in the ‘peak’ phase of the cycle. Second, the average number of copies in cells with ‘peak’ transcript level can be expressed as:

$$C^i_{peak} = A^i \times C^i_{bas} \quad (2)$$

Where *A* is the fold difference between mRNA levels at the ‘peak’ and ‘basal’ state. Combining the two equations gives us:

$$C^i_{tot} = \left(C^i_{bas} \times \frac{N_{bas}}{N_{tot}}\right) + \left(A^i \times C^i_{bas} \times \frac{N_{peak}}{N_{tot}}\right) \quad (3)$$

$$C^i_{bas} = \frac{C^i_{tot}}{\left(A^i \times \frac{N_{peak}}{N_{tot}}\right) + \frac{N_{bas}}{N_{tot}}} \quad (4)$$

$$C^i_{bas} = \frac{C^i_{tot}}{\left(A^i \times \frac{N_{peak}}{N_{tot}}\right) + \left(1 - \frac{N_{peak}}{N_{tot}}\right)} \quad (5)$$

Relationship (2) and (5) together demonstrate that average copy number/cell in cells with ‘basal’ and ‘peak’ levels of mRNA expression can be deduced from the average mRNA copy number/cell in the asynchronous population (C^i_{tot}), the fraction of cells expected to be in the phase of the cell cycle where expression peaks (N_{peak}/N_{tot}), and the amplitude of transcript level regulation (A^i).

We derived ‘basal’ and ‘peak’ copy number/cell for 241 periodic genes using amplitudes of regulation (*A*) from 6 cell-cycle time courses (Table S12) (Rustici et al., 2004). To detect genes with switch-like behavior, we took for each gene the median of the number of mRNA copies/cell derived using each 6 cell-cycle time courses (Rustici et al., 2004). Switch-like behavior was defined by the median mRNA copy number going from zone 1 (basal level) to zone 2 or 3 (peak level).

We also investigated the impact of transition kinetics between ‘basal’ and ‘peak’ expression on the deduced ‘basal’ and ‘peak’ mRNA expression levels. To do so, instead of assuming two discrete expression states (‘on’ and ‘off’), we allowed a ‘ramping’ period were mRNA expression levels gradually transit between “basal” and “peak” levels. In this case, Equation (5) becomes:

$$C_{bas}^{i} = \frac{C_{tot}^{i}}{\left(A^{i} \times \frac{N_{peak}}{N_{tot}}\right) + \left(1 - \frac{N_{peak-}N_{ramp}}{N_{tot}}\right) + R} \tag{6}$$

Where N_{ramp} is the fraction of the cell-cycle during which mRNAs gradually increase between the two expression states, and R is a ramping term defined as:

$$R = A^{i} \times \frac{N_{step}}{N_{tot}} \times \left(\frac{1}{n_{step}} + \frac{2}{n_{step}} + \frac{3}{n_{step}} + \ldots + \frac{(n_{step} - 1)}{n_{step}}\right) \tag{7}$$

Where N_{step} is the fraction of cells in a given intermediate stage, n_{step} is the number of intermediate stages. Each intermediate expression level has an expression level A^{i}/n_{step} higher than the preceding (Figure S5E). If a ‘peak’ length of 10% of the cell cycle and ramping periods lasting form 0% up to 90% of the ‘basal’ stage are considered with n_{step} = 1000, the number of genes with switch like behavior increases from 3 to a maximum of 7 for the M phase cluster, from 6 to 13 for the G1 cluster and stays equal to 0 for the S-phase cluster (Figure S5F). This analysis indicated that the number of genes categorized as showing a switch-like behavior with our model is only marginally affected by including a ramping period and that our estimate of the number of these genes is probably conservative.

Fluorescence Microscopy Analysis of Proteins with Periodic mRNA Expression

Eight strains expressing GFP-tagged proteins (National BioResource Project, Japan; Bähler et al., 1998) under control of their native promoters (Table S12) were grown in YES media at 32°C, 1 ml cells were spun down at 8,000rpm for 30 s and a 1 µl cell pellet slurry mounted onto a glass slide and mixed with 1 µl DAPI/Calcofluor suspension (1 in1000). Live cells were immediately visualized with a Zeiss Axioskop2 Plus fluorescence microscope using an EC Plan-Neofluar 63X/1.25 oil objective with FITC and DAPI filters. Five strains showed no detectable GFP signal, indicating very low protein expression; the remaining 2 strains, with GFP-tagged Cnp1 and Ark1, showed detectable GFP signals (Table S12). Expression of both proteins could only be observed in a small number of cells and was associated with features characteristic of defined cell-cycle stages as previously described (Petersen et al., 2001; Takayama et al., 2008). This analysis therefore supports the idea that the cell-cycle regulated proteins not detected in our proteome data show low expression restricted to specific cell-cycle stages (making them difficult to detect when measured in an asynchronous population).

Functional Enrichment Analyses

All analyses for functional enrichments were performed using a set of GO lists based on fission and budding yeast GOslim annotations (release September 2011). This set was complemented with a series of lists based on gene expression. Five lists of cell-cycle regulated genes were derived from (Rustici et al., 2004) and contain either all periodic genes, or periodic genes peaking in G1, S, M, or G2 phases of the cell cycle. Two lists contained genes of the core environmental stress response (CESR), either induced (”stress-related”) or repressed (”growth-related”) during stress (Chen et al., 2003). Four lists contained genes regulated upon nitrogen removal or during early, middle, or late meiosis (Mata et al., 2002). Finally 4 lists were computed in our laboratory and contained the 10% shortest and the 10% longest mRNAs, a list of transcription factors, and a list of proteins containing RNA-recognition (RRM) motifs (based on annotation available in PomBase) (Wood et al., 2012). Table S17 provides a list of the genes included in each functional category used in this study.

For the sliding-window analysis in Figure 2, all fission yeast mRNAs were ranked based on absolute expression. The level and significance of the overlap between a sliding window of 200 genes of increasing absolute expression levels and specific functional categories/gene lists were recorded. Significance of the overlaps was tested using a Fisher exact test, and p-values were corrected for multiple testing using the ‘FDR’ method.

SUPPLEMENTAL REFERENCES

Bähler, J., Wu, J.Q., Longtine, M.S., Shah, N.G., McKenzie, A., III, Steever, A.B., Wach, A., Philippsen, P., and Pringle, J.R. (1998). Heterologous modules for efficient and versatile PCR-based gene targeting in *Schizosaccharomyces pombe*. Yeast *14*, 943–951.

Beck, M., Schmidt, A., Malmstroem, J., Claassen, M., Ori, A., Szymborska, A., Herzog, F., Rinner, O., Ellenberg, J., and Aebersold, R. (2011). The quantitative proteome of a human cell line. Mol. Syst. Biol. *7*, 549.

Brusniak, M.Y., Bodenmiller, B., Campbell, D., Cooke, K., Eddes, J., Garbutt, A., Lau, H., Letarte, S., Mueller, L.N., Sharma, V., et al. (2008). Corra: Computational framework and tools for LC-MS discovery and targeted mass spectrometry-based proteomics. BMC Bioinformatics *9*, 542.

Deutsch, E.W., Mendoza, L., Shteynberg, D., Farrah, T., Lam, H., Tasman, N., Sun, Z., Nilsson, E., Pratt, B., Prazen, B., et al. (2010). A guided tour of the Trans-Proteomic Pipeline. Proteomics *10*, 1150–1159.

Elias, J.E., and Gygi, S.P. (2007). Target-decoy search strategy for increased confidence in large-scale protein identifications by mass spectrometry. Nat. Methods *4*, 207–214.

Furusawa, C., and Kaneko, K. (2003). Zipf's law in gene expression. Phys. Rev. Lett. *90*, 088102.

Kuznetsov, V.A., Knott, G.D., and Bonner, R.F. (2002). General statistics of stochastic process of gene expression in eukaryotic cells. Genetics *161*, 1321–1332.

Li, H., Ruan, J., and Durbin, R. (2008). Mapping short DNA sequencing reads and calling variants using mapping quality scores. Genome Res. *18*, 1851–1858.

Lyne, R., Burns, G., Mata, J., Penkett, C.J., Rustici, G., Chen, D., Langford, C., Vetrie, D., and Bähler, J. (2003). Whole-genome microarrays of fission yeast: characteristics, accuracy, reproducibility, and processing of array data. BMC Genomics *4*, 27.

Newman, M.E.J. (2005). Power laws, Pareto distributions and Zipf's law. Contemp. Phys. *46*, 323–351.

Petersen, J., Paris, J., Willer, M., Philippe, M., and Hagan, I.M. (2001). The S. pombe aurora-related kinase Ark1 associates with mitotic structures in a stage dependent manner and is required for chromosome segregation. J. Cell Sci. *114*, 4371–4384.

Ramsköld, D., Wang, E.T., Burge, C.B., and Sandberg, R. (2009). An abundance of ubiquitously expressed genes revealed by tissue transcriptome sequence data. PLoS Comput. Biol. *5*, e1000598.

Slater, G.S., and Birney, E. (2005). Automated generation of heuristics for biological sequence comparison. BMC Bioinformatics *6*, 31.

Takayama, Y., Sato, H., Saitoh, S., Ogiyama, Y., Masuda, F., and Takahashi, K. (2008). Biphasic incorporation of centromeric histone CENP-A in fission yeast. Mol. Biol. Cell *19*, 682–690.

Wood, V., Gwilliam, R., Rajandream, M.A., Lyne, M., Lyne, R., Stewart, A., Sgouros, J., Peat, N., Hayles, J., Baker, S., et al. (2002). The genome sequence of *Schizosaccharomyces pombe*. Nature *415*, 871–880.

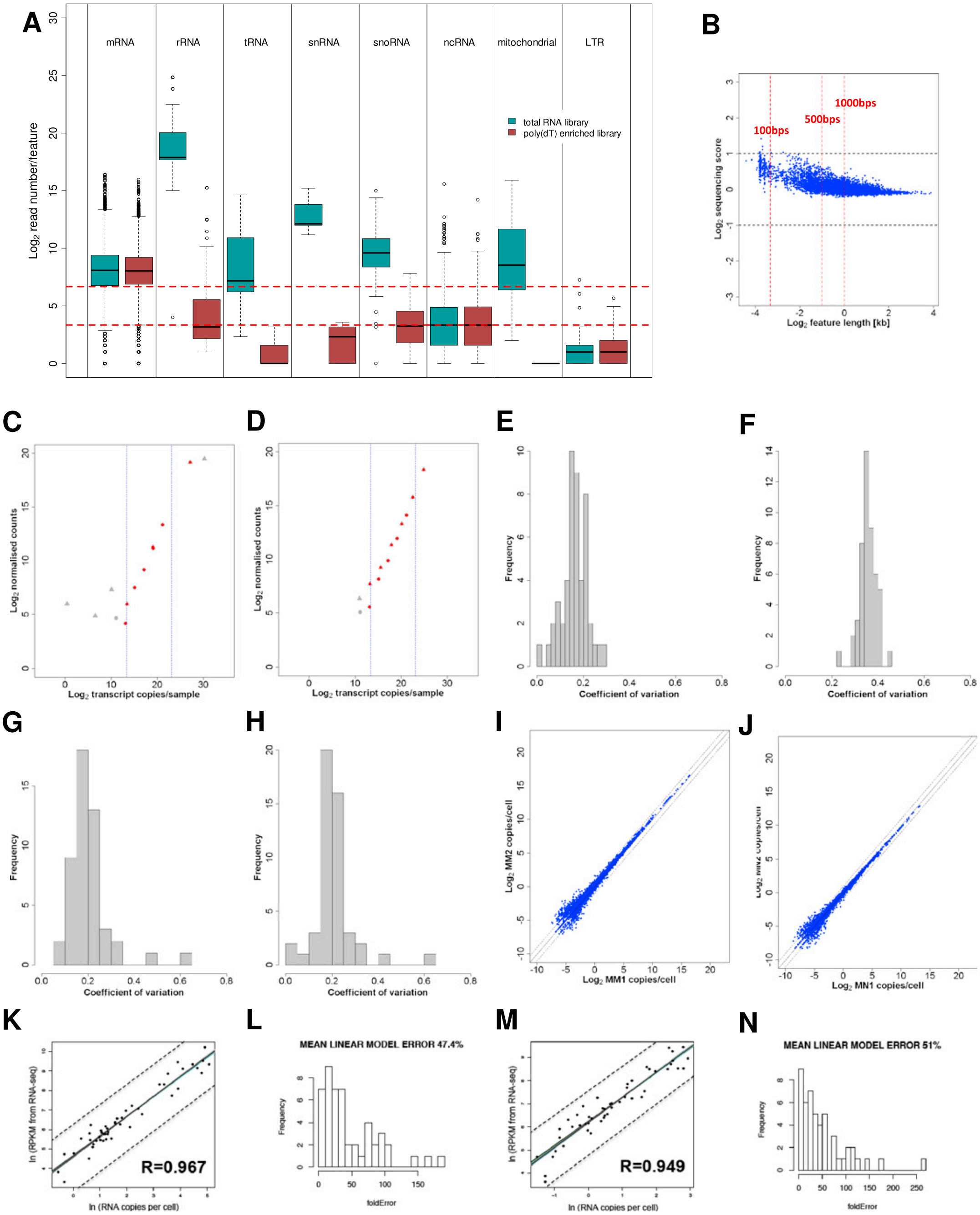

Figure S1. Analysis of Total and Poly(dT)-Enriched Transcriptomes by Strand-Specific RNA-Seq and Calibration of RNA-Seq Data Using Absolute Measurements, Related to Figure 1

Data presented in this figure are described in detail in the Extended Experimental Procedures section.

(A) Box plot of absolute reads counts in RNA-seq libraries derived from total (green) or poly(dT)-enriched (red) transcriptomes for different RNA categories. The lower and upper red lines indicate 10 and 100 sequencing reads, respectively.

(B) Plot for transcript length and the correction score derived from simulated data. The red vertical lines represent, from left to right, 100, 500, and 1000 nucleotides.

(C) Plot of copies of external nCounter controls used for calculation of absolute copy numbers of the 49 calibration mRNAs (nCounter run I). Grey circles represent external controls present in the nCounter mastermix. Grey triangles represent external controls added to the cellular extracts. The controls marked by a red dot were used for absolute copy number calculation. The blue dotted lines represent the most lowly and most highly expressed mRNAs for the 49 calibration genes, showing that the spikes used for copy number calculation support the whole dynamic range of the calibration set.

(D) Same as (B) for nCounter run II.

(E–H) Distribution of the coefficient of variations (σ/μ) of absolute copy numbers for the 49 mRNAs from the calibration set, calculated from three nCounter technical replicates split between two individual runs. (E) proliferating cells (MM1), (F) proliferating cells (MM2), (G) quiescent cells (MN1), (H) quiescent cells (MN2).

(I) Plot of mRNA copies/cell for two independent biological repeats of proliferating cells (MM1 and MM2).

(J) Plot of mRNA copies/cell for two independent biological repeats of quiescent cells (MN1 and MN2).

(K) Natural logarithm of corrected RPK scores plotted against the natural logarithm of copies per cell for 49 mRNAs quantified by nCounter for proliferating cells.

(L) Distribution of error rates determined by bootstrapping for mRNA quantities from proliferating cells.

(M) Natural logarithm of corrected RPK scores plotted against the natural logarithm of copies per cell for 49 mRNAs quantified by nCounter for quiescent cells.

(N) Distribution of error rates determined by bootstrapping for mRNA quantities from quiescent cells.

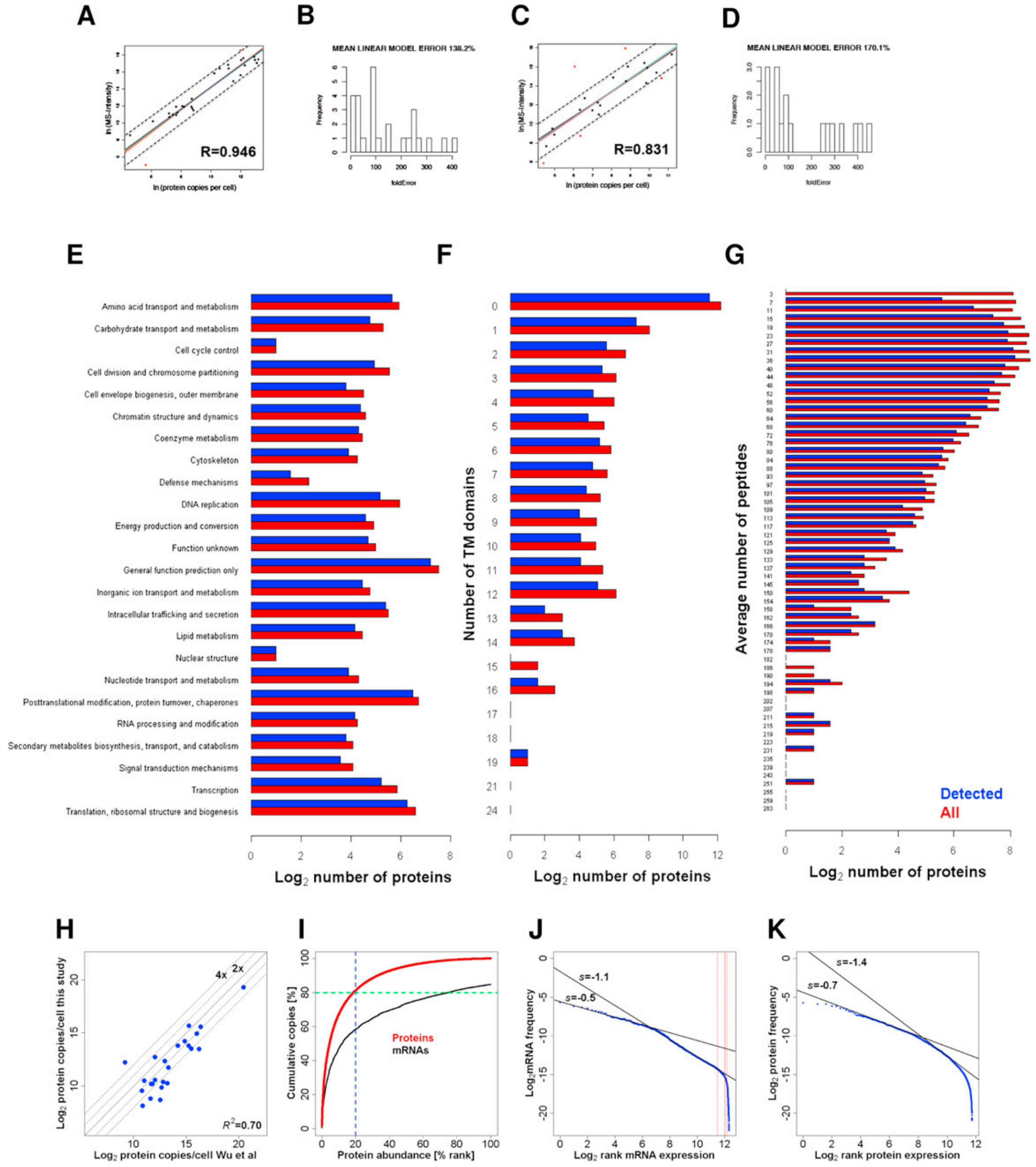

Figure S2. Calibration of Proteomics Data and Functional Properties of Fission Yeast Proteome, Related to Figure 5

(A) Natural logarithm of extracted precursor ion intensities plotted against the natural logarithm of copies per cell for 39 proteins quantified by heavy peptide standards for proliferating cells.

(B) Distribution of error rates determined by bootstrapping for protein quantities from proliferating cells.

(C) Natural logarithm of extracted precursor ion intensities plotted against the natural logarithm of copies per cell for 39 proteins quantified by heavy peptide standards for quiescent cells.

(D) Distribution of error rates determined by bootstrapping for protein quantities from quiescent cells.

(E) Distributions of Identified (blue bars) and all Database Protein Entries (red bars) for Clusters of orthologous groups (COG).

(F) Distributions of Identified (blue bars) and all Database Protein Entries (red bars) for number of transmembrane domains per protein.

(G) Distributions of Identified (blue bars) and all Database Protein Entries (red bars) for number of predicted MS-suitable peptides based on a precursor mass of 700–6000 daltons.

(H) Comparison of expression levels of 17 cytokinesis proteins in asynchronous cultures as measured in this study or in a quantitative fluorescence microscopy study (Wu and Pollard, 2005). Dotted lines represent 2 and 4 fold difference. The coefficient of determination is shown in the bottom right corner.

(I) Cumulative plot of the percentage of total protein count in proliferating cells as a function of the percentage expression rank of individual proteins (red curve), and of the percentage of total mRNA count as a function of the percentage expression rank of individual mRNAs (black curve). Blue and green lines mark 20 and 80% respectively.
(J) Log-log plots of mRNA frequencies as a function of their expression rank. Numbers indicate the exponents of selected power-law distributions shown as black curves. The red vertical lines on the left panel delimitate the three mRNA expression zones (see main text). For more information about Pareto and Zipf laws, see (Furusawa and Kaneko, 2003; Kuznetsov et al., 2002; Newman, 2005).
(K) Log-log plots of protein (right panel) frequencies as a function of their expression rank. Numbers indicate the exponents of selected power-law distributions shown as black curves. Our data thus extend Zipf's law to protein abundance.

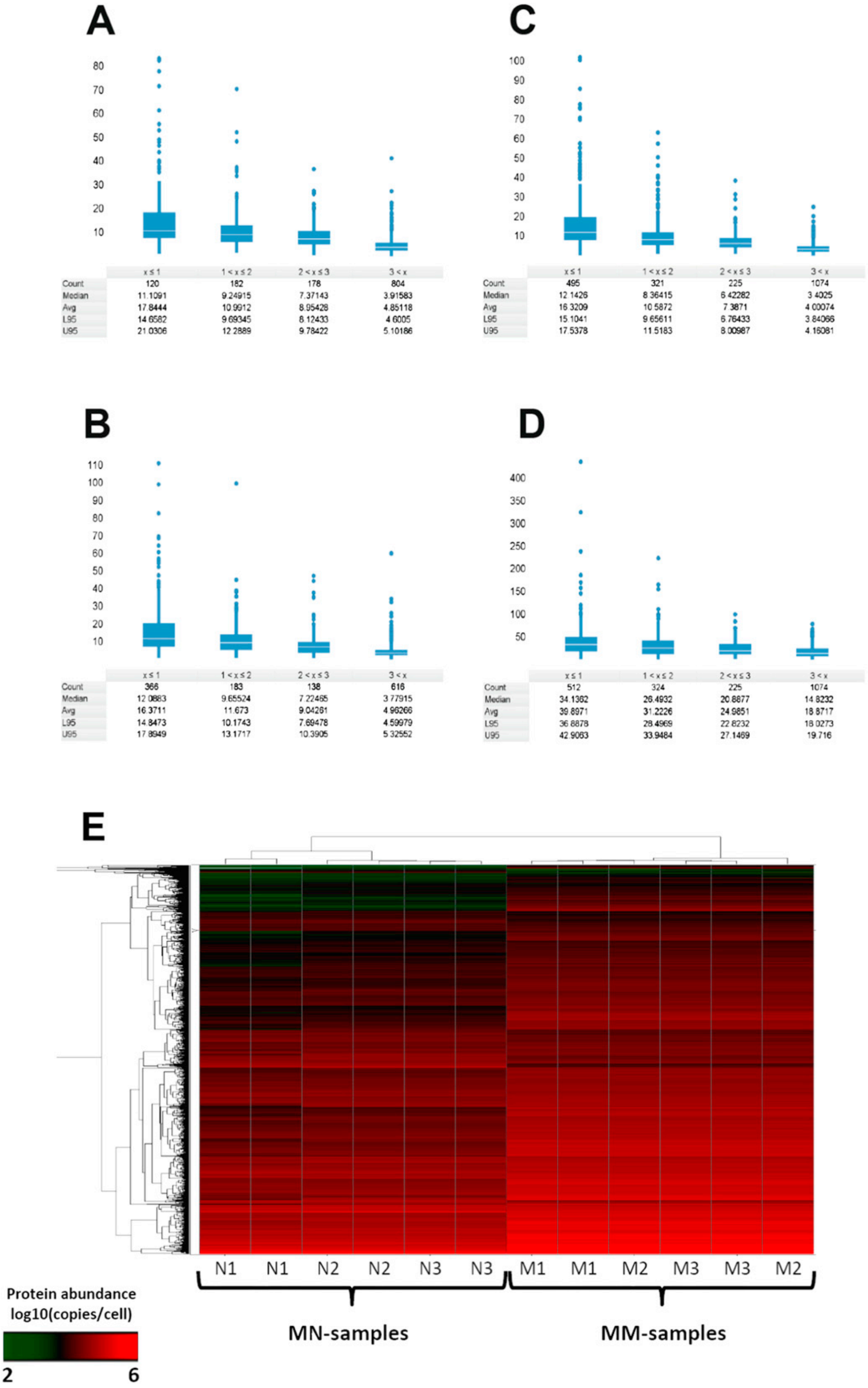

A

	x ≤ 1	1 < x ≤ 2	2 < x ≤ 3	3 < x
Count	120	182	178	804
Median	11.1091	9.24915	7.37143	3.91583
Avg	17.8444	10.9912	8.95428	4.85118
L95	14.6582	9.69345	8.12433	4.6005
U95	21.0306	12.2889	9.78422	5.10186

C

	x ≤ 1	1 < x ≤ 2	2 < x ≤ 3	3 < x
Count	495	321	225	1074
Median	12.1426	8.36415	6.42282	3.4025
Avg	16.3209	10.5872	7.3871	4.00074
L95	15.1041	9.65611	6.76433	3.84066
U95	17.5378	11.5183	8.00987	4.16081

B

	x ≤ 1	1 < x ≤ 2	2 < x ≤ 3	3 < x
Count	366	183	138	616
Median	12.0883	9.65524	7.22465	3.77915
Avg	16.3711	11.673	9.04261	4.96266
L95	14.8473	10.1743	7.69478	4.59979
U95	17.8949	13.1717	10.3905	5.32552

D

	x ≤ 1	1 < x ≤ 2	2 < x ≤ 3	3 < x
Count	512	324	225	1074
Median	34.1362	26.4932	20.8877	14.8232
Avg	39.8971	31.2226	24.9851	18.8717
L95	36.8878	28.4969	22.8232	18.0273
U95	42.9063	33.9484	27.1469	19.716

Cell

Figure S3. Technical and Biological Variability in Proteomics Data, Related to Figure 5

(A) Technical variability determined from replicate LC-MS/MS analyses for MM samples (proliferating). The median, mean, lower and upper endpoints of 95% confidence interval (L95 and U95) are displayed for proteins being quantified by 1, 2, 3 or more peptides.

(B) Expression variability (technical and biological) between the three MM biological replicates (proliferating).

(C) Technical variability determined from replicate LC-MS/MS analyses for MN samples (quiescent). The median, mean, lower and upper endpoints of 95% confidence interval (L95 and U95) are displayed for proteins being quantified by 1, 2, 3 or more peptides.

(D) Expression variability (technical and biological) between the three MN biological replicates (quiescent).

(E) Hierarchical clustering of absolute protein abundance in copies per cell (log10) for all 6 samples (3x MM, 3x MN) measured in duplicates each. The column dendrogram representing the clustering of the different samples is displayed.

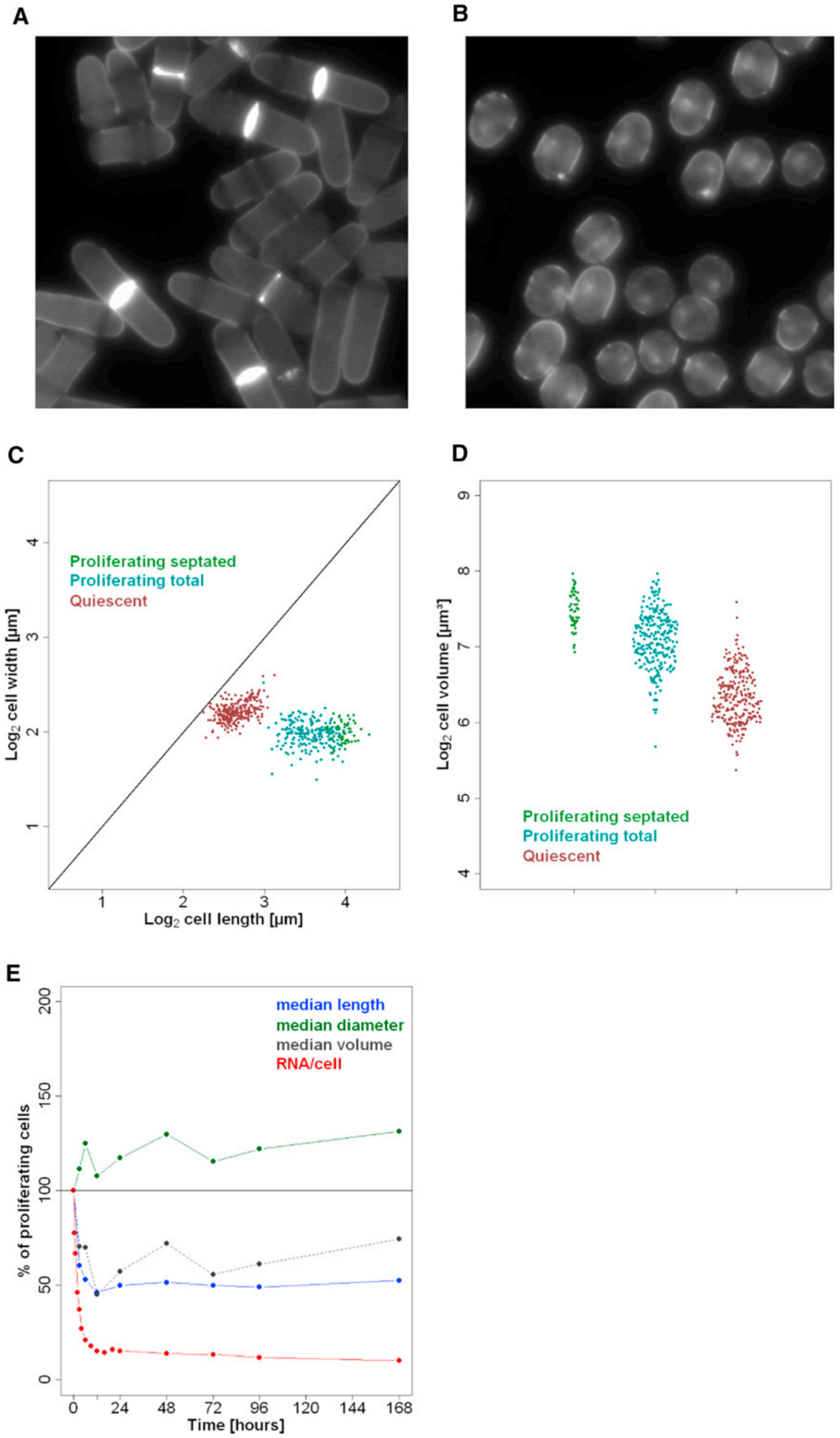

Figure S4. Cell Morphology during Proliferation and Quiescence, Related to Figure 6

(A) Proliferating fission yeast cells stained with calcofluor to highlight the division septa.

(B) As in (A) for quiescent cells, 24h after nitrogen removal.

(C) Plot of lengths and widths for 260 cells during proliferation (blue: all cells, green: septated cells), and quiescence (red).

(D) Plot for distribution of cell volume for 260 cells during proliferation (blue: all cells, green: septated cells), and quiescence (red).

(E) Plot for changes in cell length (blue), cell diameter (green), cell volume (gray) and total cellular RNA content (red), before and at multiple time points after nitrogen removal. Data are plotted as percentages of proliferating cells.

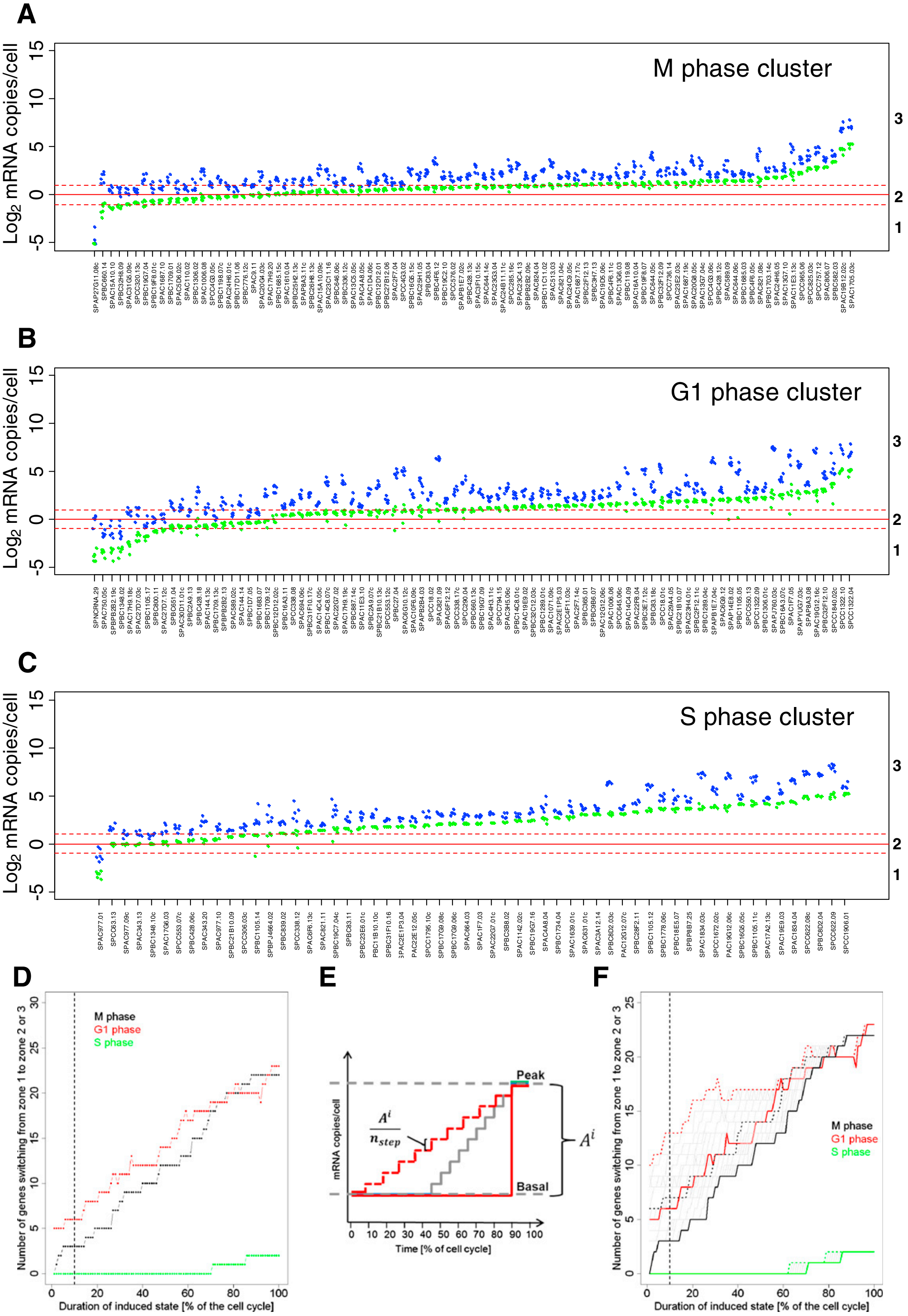
A
M phase cluster
Log2 mRNA copies/cell
3
2
1
B
G1 phase cluster
Log2 mRNA copies/cell
3
2
1
C
S phase cluster
Log2 mRNA copies/cell
3
2
1
D
M phase
G1 phase
S phase
Number of genes switching from zone 1 to zone 2 or 3
Duration of induced state [% of the cell cycle]
E
Peak
Basal
mRNA copies/cell
Time [% of cell cycle]
F
M phase
G1 phase
S phase
Number of genes switching from zone 1 to zone 2 or 3
Duration of induced state [% of the cell cycle]

Figure S5. Basal and Peak Expression of Periodic Genes, Related to Figure 3

(A) Genes from M cluster, ranked according to their median basal expression levels. The horizontal red lines delimit expression zone 2 (0.5-2 mRNA copies/cell), and the three expression zones are indicated at right.

(B) Same as (A) for genes from G1 cluster.

(C) Same as (A) for genes from S cluster.

(D) Plot of the number of genes with median basal expression switching from expression zone 1 to expression zones 2 or 3 as a function of the assumed duration of the peak phase in percent of the cell cycle for the three gene clusters (black: M phase, red: G1 phase, green: S phase). The vertical dotted line marks a 'peak' phase length of 10% of the cell cycle.

(E) Cartoon showing three example transition patterns between 'basal' and 'peak' expression levels: An instantaneous change between the two expression states (red), expression level increases during 50% (gray) or 90% (dotted red) of the non-peak window. A^i : Amplitude of periodic variation in expression for gene *i* (Rustici et al., 2004).

n_{step} : Number of intermediate states between 'basal' and 'peak' levels.

(F) Impact of ramping time on the number of genes switching from zone 1 to zones 2-3 in three clusters of periodic genes as in (D), when either no ramping (red), ramping times between 10% and 80% (gray), or 90% (dotted red) of the 'basal' phase are incorporated in the model (n_{step} = 1000). The vertical dotted line marks a 'peak' phase length of 10% of the cell cycle.

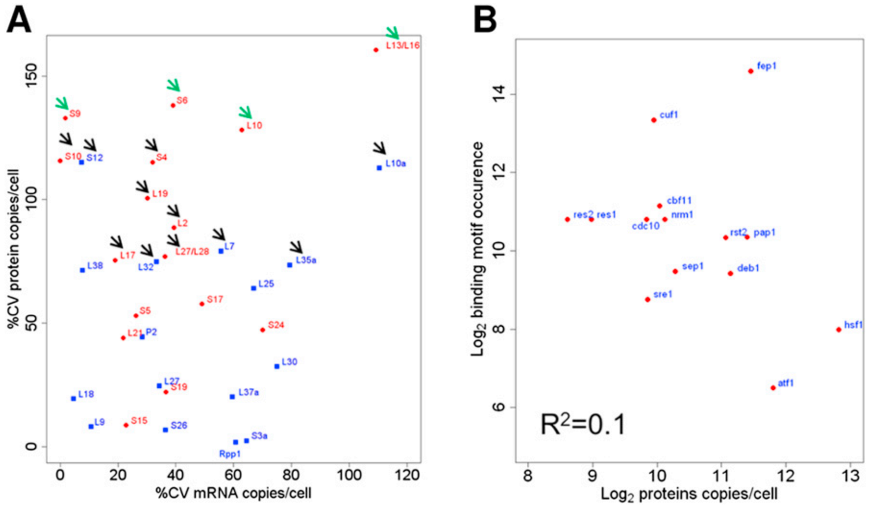

Figure S6. Additional Analyses on Ribosomal Protein Paralogs and Transcription Factors, Related to Figure 5

(A) Expression variability of ribosomal proteins. Percent coefficient of variation in mRNA and protein expression of paralog genes from different ribosomal proteins families. Red dots: families containing repeated sequences (ambiguous mapping by RNA-seq). Blue squares: families with non-repeated sequences (unambiguous mapping by RNA-seq). Dots are labeled with ribosomal protein family. Black arrows: families with over 3-fold difference in protein expression between lowest and highest expressed member. Green arrows: as black arrows but for 10-fold difference.

The median mRNA expression of single-copy ribosomal proteins is significantly higher than the median mRNA expression of duplicated ribosomal proteins (1.7-fold, $P_{wilcox} < 10^{-4}$). This patterns holds also for protein expression (1.7-fold, $P_{wilcox} < 0.02$). As most paralogs are found in two copies, this finding suggests that each paralog might contribute to about half of the ribosomes. Fission yeast ribosomes could therefore be heterogeneous complexes with respect to paralogs. However, possible technical or classification artifacts could contribute to this observation, as RNA-seq and the proteomics approach cannot unambiguously assign expression levels to paralogs with almost identical sequences. To look in more detail into paralog expression, we calculated the percent coefficient of variation (%CV) of mRNA and protein expression for each ribosomal protein family. High %CV indicates large differences in expression between paralogs. This analysis indicates that families with low %CV, where both paralogs are likely to contribute to ribosomes, can be found in cases where sequences were sufficiently diverged to permit reliable read assignment (blue squares). Moreover, some families showed vastly divergent protein expression between paralogs (arrows), suggesting either the existence of rare specialized ribosomes or extra-ribosomal functions of the lowly expressed paralogs.

(B) Comparison of TF expression levels with the occurrence of their DNA-binding motifs in the genome. Expression levels of TFs for which the DNA-binding motifs are available in PomBase were plotted against the number of their respective motifs found in the genome. Each dot represents a TF with its common name indicated in blue. It is problematic to localize true TF binding sites based on genome sequence alone, and ChIP-chip or ChIP-seq data are required to identify accurately the number of functionally relevant motifs.

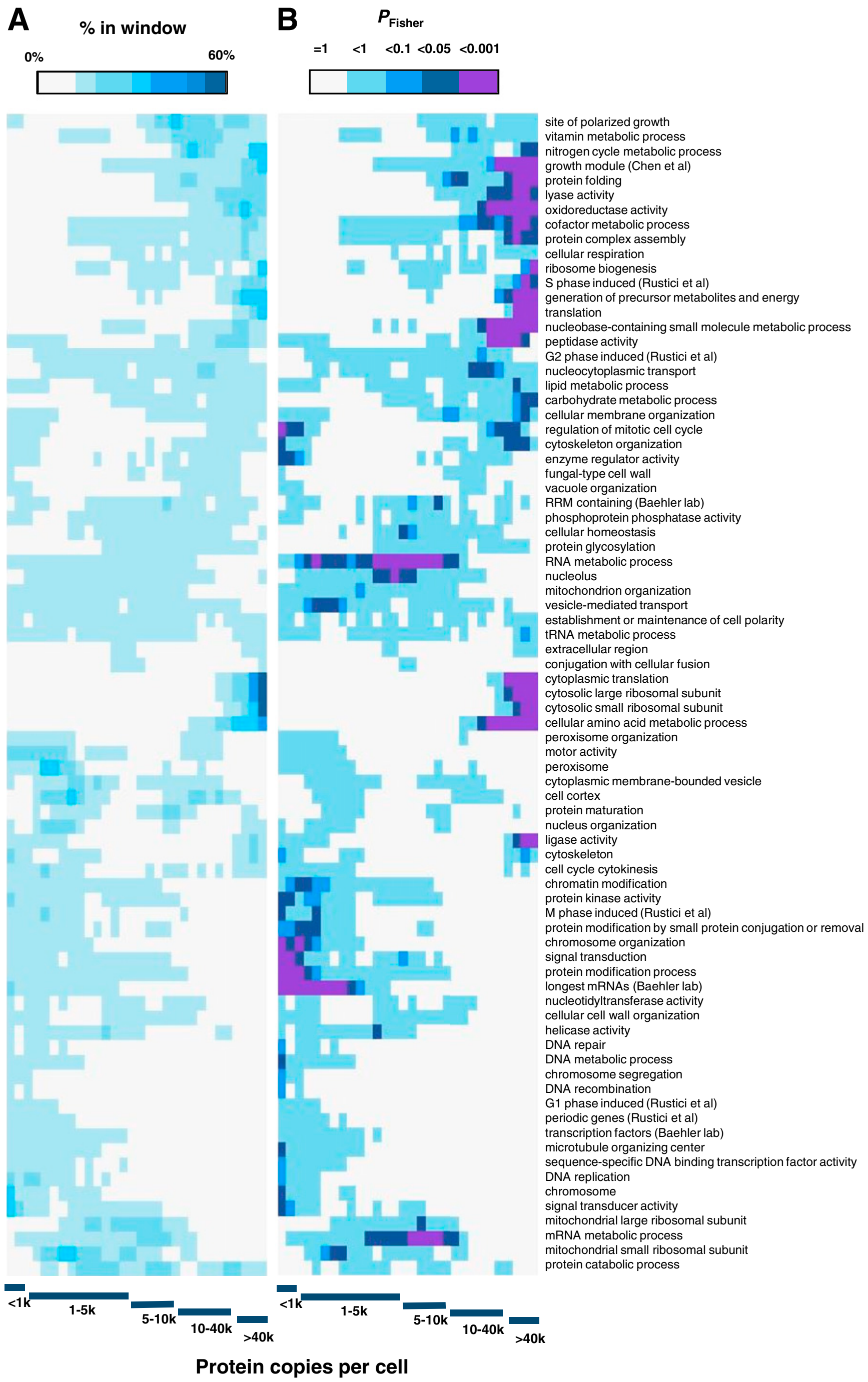

Figure S7. Functional Categories and Protein Copy Numbers, Related to Figure 5

(A) Hierarchical cluster of the percentage overlap between different functional categories and sliding windows of 200 proteins of increasing abundance. Categories with at least one window containing > 15% of the proteins in a category are plotted.

(B) Hierarchical cluster of the p-values of Fisher exact tests assessing the significance of the overlap between different functional categories and sliding windows of 200 proteins of increasing abundance.
